# TRAF6 as a potential target in advanced breast cancer: a systematic review, meta-analysis, and bioinformatics validation

**DOI:** 10.1038/s41598-023-31557-0

**Published:** 2023-03-21

**Authors:** Feier Zeng, Giovana Carrasco, Boya Li, Antonia Sophocleous, Aymen I. Idris

**Affiliations:** 1 Department of Oncology and Metabolism, Medical School, University of Sheffield, Beech Hill Road, Sheffield, S10 2RX UK; 2 Department of Life Sciences, School of Sciences, European University Cyprus, 6 Diogenes Street, 1516 Nicosia, Cyprus

**Keywords:** Bone cancer, Breast cancer, Cancer, Drug development, Target identification

## Abstract

TRAF6 has emerged as a key regulator of breast cancer (BCa). However, the TRAF family constitutes of seven members that exhibit distinct and overlapping functions. To explore which TRAF represents a potential druggable target for BCa treatment, we searched Medline, Web of Science and Scopus for relevant studies from inception to June 27, 2021. We identified 14 in vitro, 11 in vivo and 4 human articles. A meta-analysis of pharmacological studies showed that in vitro inhibition of TRAF2/4 (mean difference (MD): − 57.49, 95% CI: − 66.95, − 48.02, P < 0.00001) or TRAF6 (standard(Std.)MD: − 4.01, 95% CI: − 5.75, − 2.27, P < 0.00001) is associated with reduction in BCa cell migration. Consistently, inhibition of TRAF2/4 (MD: − 51.08, 95% CI: − 64.23, − 37.94, P < 0.00001) and TRAF6 (Std.MD: − 2.80, 95% CI: − 4.26, − 1.34, P = 0.0002) is associated with reduced BCa cell invasion, whereas TRAF2/4 inhibition (MD: − 40.54, 95% CI: − 52.83, − 28.26, P < 0.00001) is associated with reduced BCa cell adhesion. Interestingly, only inhibition of TRAF6 (MD: − 21.46, 95% CI: − 30.40, − 12.51, P < 0.00001) is associated with reduced cell growth. In animal models of BCa, administration of pharmacological inhibitors of TRAF2/4 (Std.MD: − 3.36, 95% CI: − 4.53, − 2.18, P < 0.00001) or TRAF6 (Std.MD: − 4.15, 95% CI: − 6.06, − 2.24, P < 0.0001) in mice is associated with reduction in tumour burden. In contrast, TRAF6 inhibitors (MD: − 2.42, 95% CI: − 3.70, − 1.14, P = 0.0002) reduced BCa metastasis. In BCa patients, high expression of TRAF6 (Hazard Ratio: 1.01, CI: 1.01, 1.01, P < 0.00001) is associated with poor survival rate. Bioinformatics validation of clinical and pathway and process enrichment analysis in BCa patients confirmed that gain/amplification of TRAF6 is associated with secondary BCa in bone (P = 0.0079), and poor survival rate (P < 0.05). Overall, TRAF6 inhibitors show promise in the treatment of metastatic BCa. However, low study number and scarcity of evidence from animal and human studies may limit the translation of present findings into clinical practice.

## Introduction

The TNF receptor associated factor (TRAF) family of adaptor proteins is implicated in a plethora of physiological functions, particularly inflammation and immunity^1–5^. The seven known members of the TRAF family, namely TRAF1 to 7—serve as common targets for a myriad of pro-inflammatory and immune-modulatory factors that are implicated in the regulation of oncogenic activity^1,3,4,6–11^. Given their ubiquitous expression in healthy and cancerous tissues^6,10,12–14^, a number of TRAFs have emerged as potential druggable targets in the treatment of difficult-to-treat cancers, including advanced, metastatic breast cancer^6,7^. Among TRAFs, TRAF6 is the most studied in breast cancer. A number of studies have shown that TRAF6 is highly expressed in breast cancer tumours of primary and metastatic origin^6,7^. Furthermore, TRAF6 is commonly associated with E3 ubiquitin ligase activity, as well as other homeostatic processes implicated in various aspects of hormone-dependant and triple-negative breast cancer^1,2,6,7,15^. TRAF6 (and to a lesser extent TRAF2-5) is also known to act as a point of convergence for multiple breast cancer-driver signal transduction pathways such as PI3K/AKT/mTOR, Toll-like receptor (TLR), mitogen-activated protein kinase (MAPK), NFκB, Ras/Src Family Kinases, and members of the activator protein 1 (AP-1) family^1,2,7,15,16^. Whilst these findings indicate that TRAF6 is an important regulator of breast cancer tumorigenesis and metastasis, accumulating evidence from studies in advanced breast cancer patients suggests that the expression of TRAF2 and 4 is associated with poor survival rates. Furthermore, findings from a number of in vitro and in vivo studies have shown that manipulation of TRAF2, 3 and/or 4 influences the behaviour of various breast cancer cells with different growth and metastatic abilities^6,17–22^.

Different members of the TRAF family are known to exhibit distinct and overlapping functions, and accordingly exert disparate physiological and pathophysiological effects through different mechanisms^1–5,16,23^. Thus, selecting which TRAF to study and ultimately target for the treatment of a multi-factorial and -faceted cancer such as advanced, metastatic breast cancer is a difficult challenge. We, therefore, undertook a combined systematic review, meta-analysis, and bioinformatics validation approach to examine the hypothesis that TRAF expression and modulation are associated with breast cancer progression and metastasis.

## Materials and methods

### Systematic review and meta-analysis

#### Literature search strategy

The present meta-analysis was conducted in accordance with the Preferred Reporting Items for Systematic Reviews and Meta-analyses (PRISMA) statement^24^. Briefly, we performed a comprehensive search for relevant articles in Medline, Web of Science and Scopus databases from inception to June 27, 2021. Keywords related to TRAF1-7 and breast cancer (Table S1), their combination and alternatives were used to identify articles that reported relevant studies. Articles were curated, and duplicates were identified and removed using EndNote X9 (Clarivate, London, UK).

#### Study selection

Studies that utilized animal intervention (in vivo) and in vitro models to examine the effects of pharmacological and/or genetic manipulation of TRAF1-7 on breast cancer cell behaviour were included. Human studies that reported overall and disease-specific (including metastasis-free) survival for breast cancer were included. Reviews, editorials, commentaries, case reports and abstracts were excluded. We also excluded articles that were published in a language other than English, and those that reported unspecified outcomes (Tables S1 and S2).

#### Types of interventions

Included pharmacological inhibitors of TRAF1-7 must be synthetic chemicals (e.g. TJ-M2010-2 and 6877002) or bioactive extracts (e.g. Wogonoside and plumbagin) that have been verified to exert significant reduction in the expression and activity of at least one member of the TRAF family. Genetic manipulation of TRAF1-7 must be performed using standard techniques such as shRNA, microRNA (e.g. miR146a, miR146b, miR146a/b, miRZip-892b) and others (e.g. Ei24, TLR5, CHIP) that have been verified to have significant modification in the specified gene.

#### Study outcomes

Eligible studies include a selected panel of outcomes obtained from in vitro*, *in vivo*,* and human studies (Tables S3–5). Included in vitro studies assessed cell migration by wound healing or trans-well assays, cell invasion by trans-well assay, cell proliferation by MTT (3-(4,5-dimethylthiazol-2-yl)-2,5-diphenyltetrazolium bromide), MTS (3-(4,5-dimethylthiazol-2-yl)-5-(3-carboxymethoxyphenyl)-2-(4-sulfophenyl)-2H-tetrazolium) or CCK8 (Cell Counting Kit 8) assays, colony formation by counting the numbers of 1% crystal violet stained colonies, apoptosis by using the Annexin V/PI apoptosis kit (Tables S3), respectively. Included in vivo studies assessed tumorigenesis by measuring tumour volume/weight, and metastasis by using bioluminescent imaging or immunohistochemical staining (Table S4). Included human studies assessed survival rates for breast cancer by Kaplan Meier analysis (Table S5).

#### Selection of studies

F.Z., B.L. and G.C. independently reviewed titles, abstracts and full-text of articles using predetermined inclusion and exclusion criteria. Articles excluded after review of full-text are listed in Table S2. A third reviewer (A.I.I. and/or A.S.) resolved disagreements regarding the inclusion and exclusion process.

#### Data extraction

F.Z., B.L. and G.C. extracted, curated, and analysed data from in vitro studies, and A.S and F.Z. analysed data from in vivo and human studies. A.I.I. reviewed included data. Items obtained from relevant studies include authors’ name, publication year, experimental design, sample size, outcome measures. For human studies, Hazard Ratio (HR (95%CI)) was extracted from relevant studies, or calculated according to relevant numerical values from Kaplan–Meier curve^25,26^. WebPlotDigitizer (https://apps.automeris.io/wpd/) was used to extract mean and standard deviation (SD) or standard error measurement (SEM). If mean ± SEM was reported, the SD was obtained using the formula *SEM* = *SD/√N*.

#### Data analysis

Meta-analysis was performed using Review Manager (RevMan 5). Mean difference was used as the effect measure if the same outcome/unit of measure were used, otherwise standardized (std.) mean difference was used. If heterogeneity was considered small to moderate (i.e. I^2^ < 50%), then the fixed effect analysis model was employed. The random effect analysis model was used if heterogeneity was considered high (i.e. I^2^ > 50%). For studies that involved human subjects, HR (95%CI) and number of patients were extracted from the Kaplan–Meier curve, unless HR was provided in manuscript. HR > 1 indicates poor survival rate. Ln(HR) was used as the effect measure if the same outcome and unit of measure were used in all studies included in a forest plot. Ln(HR) was calculated using the generic inverse variance method. Random effect analysis model was used if heterogeneity was high (i.e. I^2^ > 50%).

#### Quality assessment

Quality assessment of all eligible in vivo and in vitro studies were assessed using the Syrcle risk of bias^27^, and OHAT (Office of Health Assessment and Translation) risk of bias rating tool^28,29^, respectively. The following criteria were used for the Syrcle risk of bias rating: baseline characteristics, sequence generation, random housing and outcome assessment, allocation concealment, blinding of researchers and outcome assessors, incomplete outcome data, selective outcome reporting, and other sources of bias. The OHAT risk of bias rating criteria used for in vitro studies are as follows: randomization, identical experimental conditions, allocation concealment, complete outcome data, blinding of researchers, outcome assessment, exposure characterization, outcome reporting, and no other potential threats^28,29^. For human studies, the quality of eligible studies was assessed by numbers of the cases; representativeness of collected cases; TRAFs judgement criteria; and the source of HR (95%CI)^30^.

#### Certainty of evidence

The grading of recommendations assessment, development, and evaluation (GRADE) approach was used to assess certainty of the evidence from eligible human studies^31^. An adapted GRADE approach for preclinical systematic reviews was used for in vivo and in vitro experimental outcomes^32^.

#### Publication bias

No funnel plot asymmetry analysis was carried out since none of the pooled analysis included 10 or more studies^33^.

#### Microarray analysis

The normalized gene expression profiles were obtained from gene expression omnibus (GEO). The GSE14020 and GSE56493 datasets of tissue samples from metastatic breast cancer patient were used, and batch effect was corrected using BatchSever^34^. A total of 184 tissue samples were identified (bone n = 23, brain n = 22, liver n = 32, lung n = 22, lymph nodes n = 44, skin n = 22, and breast n = 19).

#### Gene expression

Copy number variation (CNVs) and gene mutations in TRAFs in samples from primary (n = 138) and metastatic (n = 282) breast cancer patients were obtained from the Metastatic Breast Cancer Project (ongoing—published to cBioportal^35,36^). Patients were separated into those who exhibited amplification (Amp), deletion (Del) and mutation (Mut) in TRAFs, and analysis was carried out using GraphPad (Prism, version 9).

#### Survival rate analysis

Kaplan–Meier survival analysis was performed to assess the overall survival (OS) in a cohort of 1951 of breast cancer patients. Data was obtained from the Molecular Taxonomy of Breast Cancer International Consortium (METABRIC)^37^. The Kaplan–Meier estimator was used to estimate the survival rate on TRAF6 expression (diploid and amplification). The log-rank test was used to compare survival rate between groups, and the hazard ratio was estimated using the Cox proportional hazards model. The statistical significance level was set at P < 0.05.

#### Functional enrichment analysis

To identify enriched biological pathways and functions associated with TRAFs, KEGG (Kyoto Encyclopedia of Genes and Genomes) and GO (Gene Ontology) enrichment analysis were performed using the STRING (Search Tool for the Retrieval of Interacting Genes/proteins) database (version 11.5)^38^. Enrichment scores were calculated as Log10(observed/expected). The minimum required interaction score was set to high confidence (0.7), and the cut-off threshold was set to have an FDR (False Discovery Rate) value of less than 0.05^39^. Bubble charts were generated using MATLAB 9.12.

### Ethics approval and consent

Data extraction and analyses were based on studies previously published on the described scientific databases and sources; thus, no ethical approval and patient consent are required.

## Results

### Articles selection

**Figure 1:**
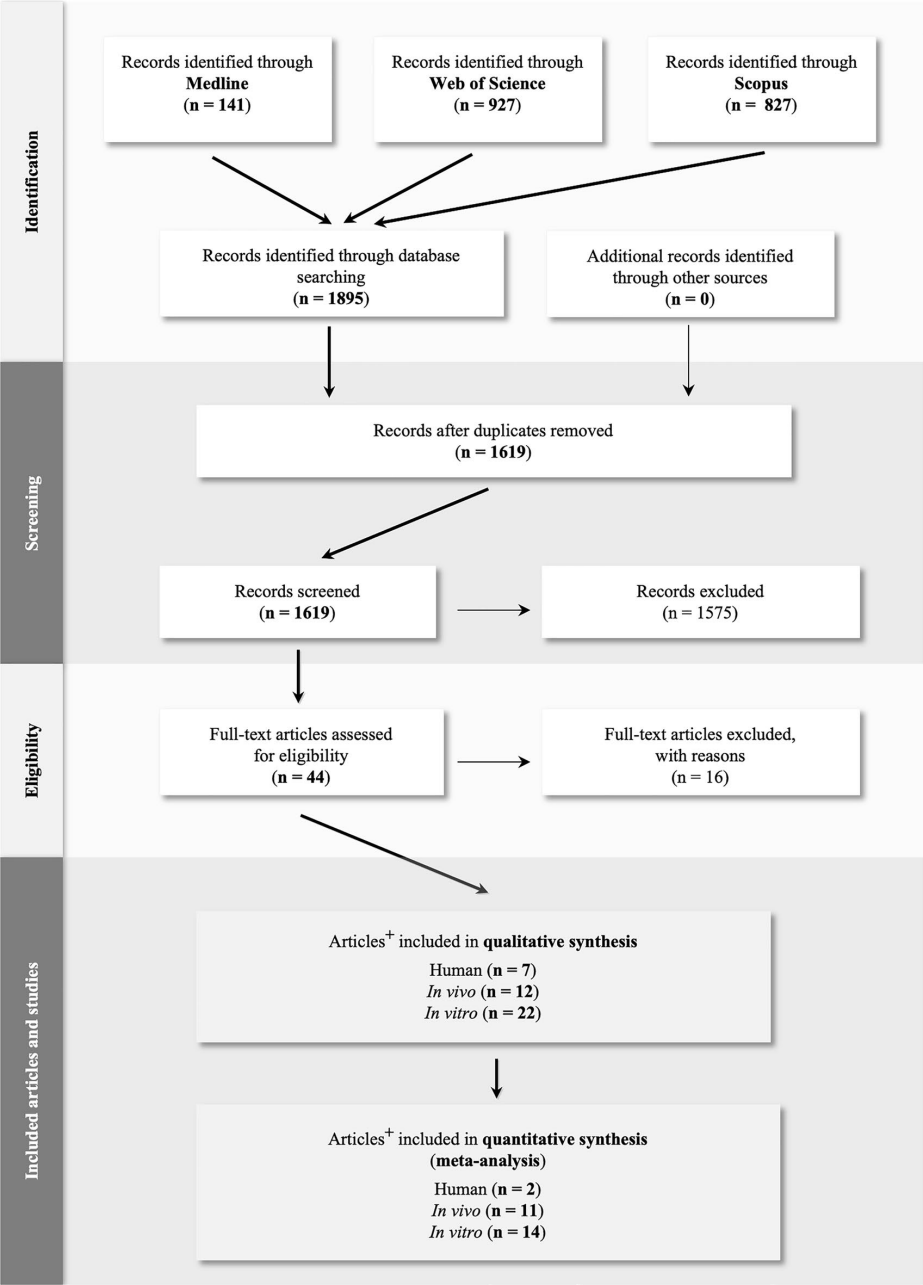
Systematic Reviews and Meta-analysis (PRISMA) flow diagram of evidence search and study selection process. ^+^ A number of included articles featured more than one study type (i.e. in vitro, in vivo and/or human studies). n denotes number of articles.

A total of 1895 articles were identified using the search strategy described in Table S1. The flow diagram that shows literature searches, selection process and study number is shown in Fig. 1. Briefly, 1575 articles were deemed irrelevant after duplicates were removed, and title and abstracts were reviewed for relevant studies. A total of 44 full-text articles were assessed for eligibility, and an additional 16 articles were excluded after full text assessment (Table S2). Articles excluded at the full-text stage together with reasons for exclusion are shown in Table S2. As shown in Fig. 1, 28 relevant articles that reported 8 human, 12 in vitro and 22 in vivo studies were included in the qualitative synthesis. Finally, 18 articles that featured 2 human, 11 in vivo and 14 in vitro studies were included in the present meta-analysis.

### Study characteristics

The 18 studies included in the quantitative analysis were published from 2011 to 2021. It is important to note that a number of included articles featured more than one in vitro, in vivo and/or human study. At least two researchers independently reviewed each article and identified relevant studies in each article included. The main characteristics of the included studies are summarized in Table 1 and Tables S3–S5.

**Table 1:** Summary the number and characteristics of included in vitro, in vivo*,* and human studies.

In vitro study characteristic (Number of studies)	In vivo study characteristic (Number of studies)	*Human* study characteristic (Number of studies)
**Intervention/modification**	**Intervention/modification**	**Intervention/modification**
Pharmacological manipulation (50)	Pharmacological manipulation (25)	Genetic association studies (7)
Genetic manipulation (55)	Genetic manipulation (12)	
**Target TRAFs**	**Target TRAFs**	**Target TRAFs**
TRAF2 (54)	TRAF2 (9)	TRAF2 (1)
TARF4 (49)	TRAF (14)	TRAF4 (3)
TRAF6 (51)	TRAF6 (15)	TRAF6 (3)
**Species**	**Species (cells)**	**Species**
Mouse (7)	Human (28)	Human (7)
Human (97)	Mouse (9)	
**Cell lines**	**Strains (mice)**	**Patient number**
MDA-MB-231 (47)	C57BL/6 (28)	TRAF2 (46)
MDA-MB-435 (7)	BALB/c (9)	TRAF4 (373)
BT474 (7)		TRAF6 (346)
BT549 (8)	**Cell lines**	
MCF7 (25)	MDA-MB-231 (22)	**Study types**
B16F10 (3)	MCF7 (6)	Kaplan–Meier survival analysis (7)
ZR-75-30 (3)	4T1 (9)	
4T1 (4)		
**Study outcomes**	**Study outcomes**	**Study outcomes**
Proliferation (23)	Tumour weight/ volume (28)	Hazzard ratio (HR (95%CI)) (4)
Migration (36)	Metastasis	Kaplan–Meier survival curve (3)
Invasion (31)	Lung metastasis (4)	
Adhesion (13)	Bone metastasis (4)	
Apoptosis (1)	Liver metastasis (1)	

### In vitro studies

As shown in Table 1 and Table S3, the included in vitro data were obtained from studies that used breast cancer cells from mouse (7 studies) and human (97 studies) to assess the effects of pharmacological (50 studies) and/or genetic (55 studies) manipulation of TRAF2 (54 studies), TRAF4 (49 studies) and TRAF6 (51 studies) on cancer cell proliferation (23 studies), migration (36 studies), invasion (31 studies), adhesion (13 studies) and apoptosis (1 study) (Tables 1 and S3). Summary of meta-analysis from these studies is shown in Table 2 and Table S6, respectively, and discussed under ‘In vitro *regulation of breast cancer cell behaviour by TRAF modulation*’.

**Table 2:** Summary of meta-analysis of included studies showing significant association of in vitro breast cancer cell behaviour and modulation of TRAF2/4/6.

Outcome		Intervention	Type of cell cultures (no. studies)	Subgroup (std.) mean difference (95% CI)	Overall (std.) mean difference (95% CI)	Statistical method	Test for heterogeneity	Test for overall effect
TRAF2	Migration	Pharmacological inhibition +	MCF7 (2) MDA-MB-231 (2) MDA-MB-435 (2)	− 62.02 [− 82.69, − 41.35] − 52.16 [− 72.11, − 32.20] − 58.25 [− 78.05, − 38.44]	− 57.49 [− 66.95, − 48.02]	Mean Difference (IV, Random, 95% CI)	Tau^2^ = 111.52; Chi^2^ = 25.49, df = 5 (P = 0.0001); I^2^ = 80%	Z = 11.91 (P < 0.00001)
		Genetic inhibition	MDA-MB-231 (2) B16F10 (1)	− 59.97 [− 88.86, − 31.09] − 24.12 [− 30.99, − 17.25]	− 46.88 [− 79.80, − 13.96]	Mean Difference (IV, Random, 95% CI)	Tau^2^ = 778.00; Chi^2^ = 30.10, df = 2 (P < 0.00001); I^2^ = 93%	Z = 2.79 (P = 0.005)
	Invasion	Pharmacological inhibition *	MCF7 (2) MDA-MB-231 (2) MDA-MB-435 (2)	− 61.41 [− 92.43, − 30.40] − 44.83 [− 68.12, − 21.53] − 47.48 [− 73.02, − 21.94]	− 51.08 [− 64.23, − 37.94]	Mean Difference (IV, Random, 95% CI)	Tau^2^ = 247.97; Chi^2^ = 63.60, df = 5 (P < 0.00001); I^2^ = 92%	Z = 7.62 (P < 0.00001)
		Genetic inhibition	MDA-MB-231 (2)	NA	− 45.54 [− 56.46, − 34.62]	Mean Difference (IV, Fixed, 95% CI)	Chi^2^ = 0.38, df = 1 (P = 0.54); I^2^ = 0%	Z = 8.17 (P < 0.00001)
		Genetic upregulation	MDA-MB-231 (3) ZR-75–30 (2)	2.65 [0.41, 4.89] 5.95 [1.70, 10.19]	3.37 [1.39, 5.35]	Std. Mean Difference (IV, Fixed, 95% CI)	Chi^2^ = 6.79, df = 4 (P = 0.15); I^2^ = 41%	Z = 3.33 (P = 0.0009)
	Proliferation	Genetic upregulation	MDA-MB-231 (1) ZR-75–30 (1)	2.61 [2.10, 3.12] 3.34 [2.98, 3.70]	3.00 [2.28, 3.71]	Mean Difference (IV, Random, 95% CI)	Tau^2^ = 0.22; Chi^2^ = 5.19, df = 1 (P = 0.02); I^2^ = 81%	Z = 8.23 (P < 0.00001)
	Adhesion	Pharmacological inhibition ^£^	MCF7 (2) MDA-MB-231 (2) MDA-MB-435 (2) BT474 (2)	− 58.48 [− 76.86, − 40.11] − 42.59 [− 62.58, − 22.60] − 41.32 [− 62.61, − 20.02] − 19.78 [− 30.75, − 8.82]	− 40.54 [− 52.83, − 28.26]	Mean Difference (IV, Random, 95% CI)	Tau^2^ = 287.52; Chi^2^ = 83.94, df = 7 (P < 0.00001); I^2^ = 92%	Z = 6.47 (P < 0.00001)
TRAF4	Migration	Pharmacological inhibition +	MCF7 (2) MDA-MB-231 (2) MDA-MB-435 (2)	− 62.02 [− 82.69, − 41.35] − 52.16 [− 72.11, − 32.20] − 58.25 [− 78.05, − 38.44]	− 57.49 [− 66.95, − 48.02]	Mean Difference (IV, Random, 95% CI)	Tau^2^ = 111.52; Chi^2^ = 25.49, df = 5 (P = 0.0001); I^2^ = 80%	Z = 11.91 (P < 0.00001)
		Genetic inhibition	MCF7 (1) MDA-MB-231 (2)	− 309.10 [− 358.84, − 259.36] − 459.60 [− 523.02, − 396.18]	− 407.97 [− 525.57, − 290.37]	Mean Difference (IV, Random, 95% CI)	Tau^2^ = 8896.64; Chi^2^ = 13.71, df = 2 (P = 0.001); I^2^ = 85%	Z = 6.80 (P < 0.00001)
		Genetic upregulation	MCF7 (3)	NA	− 89.52 [− 139.93, − 39.12]	Mean Difference (IV, Random, 95% CI)	Tau^2^ = 1728.25; Chi^2^ = 16.94, df = 2 (P = 0.0002); I^2^ = 88%	Z = 3.48 (P = 0.0005)
	Invasion	Pharmacological inhibition *	MCF7 (2) MDA-MB-231 (2) MDA-MB-435 (2)	− 61.41 [− 92.43, − 30.40] − 44.83 [− 68.12, − 21.53] − 47.48 [− 73.02, − 21.94]	− 51.08 [− 64.23, − 37.94]	Mean Difference (IV, Random, 95% CI)	Tau^2^ = 247.97; Chi^2^ = 63.60, df = 5 (P < 0.00001); I^2^ = 92%	Z = 7.62 (P < 0.00001)
		Genetic inhibition	MDA-MB-231 (4)	NA	− 7.00 [− 10.05, − 3.96]	Mean Difference (IV, Random, 95% CI)	Tau^2^ = 6.67; Chi^2^ = 38.27, df = 3 (P < 0.00001); I^2^ = 92%	Z = 4.51 (P < 0.00001)
	Adhesion	Pharmacological inhibition ^£^	MCF7 (2) MDA-MB-231 (2) MDA-MB-435 (2) BT474 (2)	− 58.48 [− 76.86, − 40.11] − 42.59 [− 62.58, − 22.60] − 41.32 [− 62.61, − 20.02] − 19.78 [− 30.75, − 8.82]	− 40.54 [− 52.83, − 28.26]	Mean Difference (IV, Random, 95% CI)	Tau^2^ = 287.52; Chi^2^ = 83.94, df = 7 (P < 0.00001); I^2^ = 92%	Z = 6.47 (P < 0.00001)
TRAF6	Migration	Pharmacological inhibition	BT549 (3) MCF7 (1) MDA-MB-231 (4) MDA-MB-231-BT (4)	− 5.60 [− 11.76, 0.56] − 9.20 [− 18.23, − 0.17] − 6.28 [− 11.63, − 0.93] − 3.21 [− 5.33, − 1.08]	− 4.01 [− 5.75, − 2.27]	Mean Difference (IV, Fixed, 95% CI)	Tau^2^ = 2.56; Chi^2^ = 16.53, df = 11 (P = 0.12); I^2^ = 33%	Z = 4.52 (P < 0.00001)
		Genetic inhibition	MCF7 (3)	NA	− 0.32 [− 0.65, 0.01]	Mean Difference (IV, Random, 95% CI)	Tau^2^ = 0.08; Chi^2^ = 476.58, df = 2 (P < 0.00001); I^2^ = 100%	Z = 1.92 (P = 0.05)
		Genetic upregulation	MCF7 (3)	NA	0.25 [0.23, 0.27]	Mean Difference (IV, Fixed, 95% CI)	Chi^2^ = 1.85, df = 2 (P = 0.40); I^2^ = 0%	Z = 3.14 (P = 0.002)
	Invasion	Pharmacological inhibition	BT549 (3) MCF7 (1) MDA-MB-231 (4) MDA-MB-231-BT (4)	− 5.78 [− 12.04, 0.47] − 9.50 [− 18.82, − 0.19] − 5.62 [− 10.34, − 0.90] − 1.36 [− 2.53, − 0.19]	− 2.80 [− 4.26, − 1.34]	Mean Difference (IV, Fixed, 95% CI)	Tau^2^ = 2.23; Chi^2^ = 19.22, df = 11 (P = 0.06); I^2^ = 43%	Z = 3.76 (P = 0.0002)
	Proliferation	Pharmacological inhibition	BT549 (4) MCF7 (4) MDA-MB-231 (8)	− 5.78 [− 12.04, 0.47] − 9.50 [− 18.82, − 0.19] − 5.62 [− 10.34, − 0.90]	− 21.46 [− 30.40, − 12.51]	Mean Difference (IV, Random, 95% CI)	Tau^2^ = 321.80; Chi^2^ = 677.01, df = 15 (P < 0.00001); I^2^ = 98%	Z = 4.70 (P < 0.00001)
		Genetic upregulation	MCF7 (1) 4T1 (3)	0.12 [0.09, 0.15] 0.38 [0.24, 0.52]	0.31 [0.12, 0.51]	Mean Difference (IV, Random, 95% CI)	Tau^2^ = 0.04; Chi^2^ = 158.59, df = 3 (P < 0.00001); I^2^ = 98%	Z = 3.19 (P = 0.001)

The analysis shows the mean difference or standard mean difference of different studies comparing the effects of the TRAF6 pharmacological inhibition, genetic inhibition or upregulation on cell migration as assessed by trans-well assay or wound healing assay, cell invasion as assessed by trans-well assay and proliferation as assessed by cell viability assay. Std., standardised; IV, inverse-variance weighting; NA, not applicable. ^+ ^Refers to studies using the same intervention which inhibit both TRAF2 and 4 on cell invasion, *refers to studies using the same intervention which inhibit both TRAF2 and 4 on cell invasion, £ intervention modulated the activity of both TRAF2 and TRAF4.

### In vivo studies

The included in vivo data were obtained from studies that tested the effects of pharmacological (25 studies) or genetic manipulation (12 studies) of TRAF2 (9 studies), TRAF4 (14 studies) and TRAF6 (15 studies) on tumour burden (weight/volume, 27 studies) and metastasis (lung, 4 studies; bone, 4 studies; liver, 1 study) using histology and bioluminescence imaging (Tables 1 and S4). Summary of meta-analysis from these studies is shown in Table 3 and Table S7, and discussed under ‘In vivo* regulation of breast cancer tumorigenesis and metastasis by TRAF modulation*’.

**Table 3:** Summary of meta-analysis of included studies showing significant association of in vivo tumour burden and overt metastasis with modulation of TRAF2/4/6.

Outcome		Intervention	Type of cell cultures (no. studies)	Subgroup (std.) mean difference (95% CI)	Overall (std.) mean difference (95% CI)	Statistical method	Test for heterogeneity	Test for overall effect
TRAF2	Tumorigenesis	Pharmacological inhibition *	MDA-MB-231 (2)	NA	− 3.36 [− 4.53, − 2.18]	Std. Mean Difference (IV, Fixed, 95% CI)	Chi^2^ = 0.00, df = 1 (P = 0.95); I^2^ = 0%	Z = 5.61 (P < 0.00001)
		Genetic upregulation	MDA-MB-231 (2)	NA	5.81 [3.91, 7.72]	Mean Difference (IV, Fixed, 95% CI)	Chi^2^ = 0.83, df = 1 (P = 0.36); I^2^ = 0%	Z = 5.98 (P < 0.00001)
TRAF4	Tumorigenesis	Pharmacological inhibition *	MDA-MB-231 (2)	NA	− 3.36 [− 4.53, − 2.18]	Std. Mean Difference (IV, Fixed, 95% CI)	Chi^2^ = 0.00, df = 1 (P = 0.95); I^2^ = 0%	Z = 5.61 (P < 0.00001)
	Metastasis	Genetic inhibition	MDA-MB-231 (1) MDA-MB-231 (2)	− 3.21 [− 4.21, − 2.21] − 1.68 [− 3.00, − 0.37]	− 2.65 [− 3.45, − 1.85]	Mean Difference (IV, Fixed, 95% CI)	Chi^2^ = 3.27, df = 2 (P = 0.19); I^2^ = 39%	Z = 6.51 (P < 0.00001)
TRAF6	Tumorigenesis	Pharmacological inhibition	MDA-MB-231 (2) MCF7 (2) 4T1 (2)	− 4.42 [− 6.16, − 2.68] − 7.32 [− 9.97, − 4.67] − 1.93 [− 3.59, − 0.27]	− 4.15 [− 6.06, − 2.24]	Std. Mean Difference (IV, Random, 95% CI)	Chi^2^ = 23.86, df = 5 (P = 0.0002); I^2^ = 79%	Z = 4.25 (P < 0.0001)
		Genetic upregulation	MDA-MB-231 (1) 4T1 (2)	4.63 [1.74, 7.51] 6.97 [4.06, 9.89]	5.79 [3.74, 7.84]	Std. Mean Difference (IV, Fixed, 95% CI)	Chi^2^ = 1.36, df = 2 (P = 0.51); I^2^ = 0%	Z = 5.53 (P < 0.00001)
	Metastasis	Pharmacological inhibition	4T1 (2)	NA	− 2.42 [− 3.70, − 1.14]	Mean Difference (IV, Random, 95% CI)	Tau^2^ = 0.70; Chi^2^ = 5.57, df = 1 (P = 0.02); I^2^ = 82%	Z = 3.69 (P = 0.0002)

The analysis shows the mean difference or standard mean difference of different studies comparing the effects of the TRAF2/4/6 pharmacological inhibition, genetic inhibition or upregulation on tumour (weight (gram)/volume, %) and overt metastasis (histology and bioluminescence imaging). Std., standardised; IV, inverse-variance weighting; NA, not applicable. * intervention modulated the activity of both TRAF2 and TRAF4.

### Human studies

The included human data were obtained from studies that examined the association between survival rate and TRAF2 expression (1 study), TRAF4 expression (3 studies) or TRAF6 expression (2 studies) (Tables 1 and S5). Sample size varied; four studies had fewer than 200 patients, three studies had more than 200 patients. All articles featured Kaplan–Meier survival curves and provided sample size: 4 studies provided HR (95%CI) and Kaplan-Merrier survival curves, and 3 studies provided Kaplan-Merrier survival curves and thus HR (95%CI) was estimated as previously described^26^.

### Quality assessment

Risk of bias for in vitro studies is shown in Fig. S2. Out of the 9 criteria only ‘blinding of research personnel during the study’ (criterion 4) was scored as ‘Probably high risk’ for in vitro studies. However, this was of no concern since it is rather uncommon for researchers to be blinded when performing in vitro experiments. Amongst the remaining criteria, 8 articles were considered ‘Probably low risk’ for criterion 6 (Exposure characterization^40–47^), all studies were considered ‘Probably low risk’ for criterion 5 (Missing outcome data) and criterion 7 (Outcome assessment). The overall risk of bias for in vitro studies was ‘probably low’ to ‘definitely low’, and thus no study was excluded solely based on their quality. Risk of bias for in vivo studies was assessed using the Syrcle tool^27^ (Fig. S1). Out of the 10 items, 4 scored as ‘high risk’ for most in vivo studies. These were item 1—Sequence generation, item 3—Allocation concealment, item 5—Blinding of researchers, and item 7—Blinding of outcome assessors. Although these quality items are imperative for high quality clinical studies, we believe that it is rather uncommon for in vivo studies to fulfil them; thus their prevalence was expected. Furthermore, 3 items scored as ‘unclear’ for a number of in vivo studies. These were item 1 – Sequence generation, 6—Random outcome assessment, and item 8—Incomplete outcome data. Once again, these were of no particular concern. If we exclude the 4 items that most articles scored ‘high risk’ (items 1, 3, 5 and 7) and the 3 items that most articles scored ‘unclear’ (items 1, 6 and 8), all articles indicated an overall high quality and hence were not excluded based solely on their quality. The overall quality of the included human studies is high apart from 1 study which was considered of medium quality due to a small sample size (Table S9)^30^.

### Narrative synthesis

Data in studies from 14 articles were considered too heterogeneous to pool or not reported in a format suitable for pooling. These articles were included in the narrative synthesis (Table 5). A number of these articles featured more than one in vitro, in vivo and/or human studies.

### Certainty of evidence

The overall quality of outcomes of in vitro and in vivo studies was judged as follows: (A) very low for in vitro cell proliferation, migration, invasion, and adhesion; (B) very low for in vivo tumour volume, bone metastasis, liver metastasis; (C) low for in vivo tumour weight and lung metastasis. For human studies, the overall quality of outcomes was judged very low due to imprecision and small sample size.

## Meta-analysis of outcomes

### In vitro studies

First, we evaluated the association between pharmacological and genetic modulation of TRAF1-7 with breast cancer cell proliferation, migration, invasion, adherence, and apoptosis in vitro. Using the aforementioned search strategy, we identified 105 individual studies from 28 relevant articles that tested the effects of pharmacological (50 studies) and genetic (55 studies) manipulation of TRAF2 (54 studies), TARF4 (49 studies) or TRAF6 (51 studies) on the in vitro proliferation (23 studies), migration (36 studies), invasion (31 studies), adhesion (13 studies) and apoptosis (1 study) of human (97 studies—MDA-MB-231, 47 studies; MDA-MB-435, 7 studies; BT474, 7 studies; BT549, 8 studies; MCF-7, 25 studies; B16F10, 3 studies) and mouse (7 studies—ZR-75-30, 3 studies and 4T1, 4 studies) breast cancer cells (Tables 1 and 2).

### In vitro regulation of breast cancer cell migration by TRAF2/4/6

Meta-analysis of included studies that tested the effects of TRAF1-7 modulation on migration of breast cancer cells in vitro (Table 2) showed that pharmacological inhibition of TRAF2, TRAF4 (6 studies, mean difference − 57.49, 95% CI − 66.95, − 48.02, Z score 11.91 (P < 0.00001)), or TRAF6 (12 studies, Std. mean difference − 4.01, 95% CI − 5.75, − 2.27 Z score 4.52 (P < 0.00001)) is associated with significant reduction in the ability of human hormone-dependent MCF-7 and triple-negative MDA-MB-435, BT549 and MDA-MB-231-BT breast cancer cells to migrate in vitro (Table 2). Consistent with findings from these pharmacological studies, genetic inhibition of TRAF2 in MDA-MB-231 and B16F10 (3 studies, mean difference − 46.88, 95% CI − 79.80, − 13.96, Z score 2.79 (P = 0.005)), TRAF4 in MDA-MB-231 and MCF-7 (3 studies, mean difference − 407.97, 95% CI − 525.57, − 290.37, Z score 6.80 (P < 0.00001)), or TRAF6 in MCF-7 (3 studies, mean difference − 0.32, 95% CI − 0.65, 0.01, Z score 1.92 (P = 0.05)) is associated with significant reduction in cell migration in vitro (Table 2). Conversely, genetic upregulation of TRAF4 in MCF-7 (3 studies, mean difference − 89.52, 95% CI − 139.93, − 39.12, Z score 3.48 (P = 0.0005)), or TRAF6 in MCF-7 (3 studies, mean difference 0.25, 95% CI 0.23, 0.27, Z score 3.14 (P = 0.002)) is associated with significant increase in cell migration in vitro (Table 2). Based on findings from pooled studies, we conclude that inhibition of TRAF2, 4 and/or 6 is associated with reduced in vitro migration of the hormone-dependent and triple negative breast cancer cells described. A review of studies that were considered too heterogeneous to pool or not reported in a format suitable for pooling (Table 1) confirmed that TRAF6 expression is associated with in vitro motility of the hormone-dependent MCF-7 and triple-negative MDA-MB231 breast cancer cells^48^.

### In vitro regulation of breast cancer cell invasion by TRAF2/4/6

Analysis of pooled in vitro studies showed that pharmacological inhibition of TRAF2 and 4 (6 studies, mean difference − 51.08, 95% CI − 64.23, − 37.94, Z score 7.62 (P < 0.00001)), or TRAF6 (12 studies, Std. mean difference − 2.80, 95% CI − 4.26, − 1.34, Z score 3.76 (P = 0.0002)) is associated with significant reduction in the in vitro invasion of human MCF-7 and triple-negative MDA-MB-435 and BT549 breast cancer cells (Table 2). Consistently, genetic inhibition of TRAF2 in MDA-MB-231 (3 studies, mean difference − 45.54, 95% CI − 56.46, − 34.62, Z score 8.17 (P < 0.00001)) and TRAF4 in MDA-MB-231 (4 studies, mean difference − 7.00, 95% CI − 10.05, − 3.96, Z score 4.51 (P < 0.00001)) is associated with significant reduction in cell invasion in vitro (Table 2). Conversely, genetic upregulation of TRAF2 in MDA-MB-231 and ZR-75-30 (5 studies, Std. mean difference 3.37, 95% CI 1.39, 5.35, Z score 3.33 (P = 0.0009)) is associated with significant reduction in cell invasion in vitro (Table 2). Thus, TRAF2, 4 and 6 regulate the in vitro invasion of the hormone-dependent and triple-negative breast cancer cells described. Findings from non-pooled papers (Table 5) partially complement this and confirm that TRAF6, but not TRAF2 or 4, regulates the invasion of MCF-7 and MDA-MB231 breast cancer cells in vitro^48^, and indirectly enhances the interaction between tumour-associated fibroblasts and breast cancer cells in co-culture models^49^. In contrast to the aforementioned pro-migratory and pro-invasive roles of TRAF2 in breast cancer, evidence from non-pooled study by Sirinian et al.^50^ suggest that TRAF2 directly interacts with an isoform of the RANK receptor termed RANK-c to inhibit the migration and invasion of MDA-MB-231 and SKBR3 breast cancer cells in vitro, and to reduce the metastatic abilities of a clone of SKBR3 breast cancer cells in mice.

### In vitro regulation of breast cancer cell adherence by TRAF2/4

Analysis of pooled in vitro studies showed that pharmacological inhibition of TRAF2/4 (8 studies, mean difference − 40.54, 95% CI − 52.83, − 28.26, Z score 6.47 (P < 0.00001)) is associated with significant reduction in the in vitro adherence of human hormone-dependent MCF-7 and the triple-negative MDA-MB-231, MDA-MB-435 and BT549 breast cancer cells (Table 2).

### In vitro regulation of breast cancer cell proliferation by TRAF6

Meta-analysis of pooled in vitro studies that examined breast cancer cell survival and apoptosis showed that pharmacological inhibition of TRAF6 (16 studies, mean difference − 21.46, 95% CI − 30.40, − 12.51, Z score 4.70 (P < 0.00001)) is associated with significant reduction in the ability of the triple-negative human breast cancer cells BT549, MCF-7, MDA-MB-231 to proliferate in vitro (Table 2). Analysis of pooled studies that examined genetic manipulation of TRAF1-7 showed that knockdown of TRAF4 (4 studies, mean difference − 7.00, 95% CI − 10.05, − 3.96, Z score 4.51 (P < 0.00001)) is associated with significant reduction in MDA-MB-231 cell invasion in vitro (Table 2). Conversely, genetic upregulation of TRAF2 in MDA-MB-231 and ZR-75-30 (2 studies, mean difference 3.00, 95% CI 2.28, 3.71, Z score 8.23 (P < 0.00001)) and TRAF6 in human MCF-7 and mouse 4T1 (4 studies, mean difference 0.31, 95% CI 0.12, 0.51, Z score 3.19 (P = 0.001)) is associated with an increase in cell proliferation in vitro (Table 2). In broad agreement with these findings, we also found evidence from non-pooled studies to indicate that exposure of MCF-7 and MDA-MB-231 cells to TJ-M2010-2, an inhibitor of MyD88 homodimerization, reduced TRAF6 expression and caused apoptotic cell death^47^. Similarly, TRAF6 inhibition was also found to be associated with the anti-proliferative and pro-apoptotic effects of miR-146a/b in human MCF-7 cells^45,51^. Although TRAF1 has been found to promote cell survival and death^6^, Wang et al.^52^ showed that its over-expression in MCF-7 cells failed to protect these cells against the anti-proliferative and pro-apoptotic effects of the chemotherapeutic agent paclitaxel. Thus, we conclude that TRAF2/4/6 modulation is associated with altered in vitro behaviour of the hormone-dependent and triple-negative breast cancer cells described.

## In vivo studies

As shown in Table 1, we identified 37 studies that tested the effects of pharmacological (25 studies) and genetic (12 studies) manipulation of TRAF2 (9 studies), TRAF4 (14 studies) or TRAF6 (15 studies) on tumour weight/volume (28 studies) and metastasis to the lung (3 studies), skeleton (4 studies) and liver (1 studies) in rodents bearing human (28 studies: MDA-MB-231, 22 studies and MCF-7, 6 studies) or mouse 4T1 (9 studies) breast cancer cells.

### In vivo regulation of breast cancer tumour burden by TRAF2/4/6

Analysis of pooled in vivo studies showed that administration of inhibitors of TRAF2 and TRAF4 (2 studies, Std. mean difference − 3.36, 95% CI − 4.53, − 2.18, Z score 5.61 (P < 0.00001)), or TRAF6 (6 studies, Std. mean difference − 4.15, 95% CI − 6.06, − 2.24, Z score 4.25 (P < 0.0001)) is associated with significant reduction in tumour weight and volume in female rodents bearing the human triple-negative MDA-MB-231 (TRAF2, TRAF4 and TRAF6) and mouse 4T1 (TRAF6) breast cancer cells (Table 3). Conversely, genetic upregulation of TRAF2 in MDA-MB-231 (2 studies, mean difference 5.81, 95% CI 3.91, 7.72, Z score 5.98 (P < 0.00001)), and TRAF6 in MDA-MB-231 or 4T1 (3 studies, Std. mean difference 5.79, 95% CI 3.74, 7.84, Z score 5.53 (P < 0.00001)) are associated with significant increase in tumour weight and volume in mice (Table 3).

### In vivo regulation of breast cancer metastasis by TRAF4/6

Our meta-analysis also included studies that examined the effects of TRAF1-7 manipulation on overt metastases in female mice bearing human and mouse breast cancer cells. As shown in Table 3, pharmacological inhibition of TRAF6 (2 studies, mean difference − 2.42, 95% CI − 3.70, − 1.14, Z score 3.69 (P = 0.0002) is associated with significant reduction in bone metastasis in rodents bearing an osteotropic clone of 4T1 cells. Consistently, genetic inhibition of TRAF4 in human MDA-MB-231 cells (3 studies, mean difference − 2.65, 95% CI − 3.45, − 1.85, Z score 6.51 (P < 0.00001)) is also associated with significant reduction in bone (2 studies) and lung metastases (Table 2). In contrast, a review of non-pooled studies (Table 5) revealed that TRAF3 activity is associated with an anti-metastatic effect^20^. Moreover, Liu et al.^20^ showed that the osteoprotective effects of osteoclastic miR-214 in mice bearing MDA-MB-231 cells was accompanied by also significant increase in intraosseous level of TRAF3.

### Human studies

As shown in Table 1, our research identified 7 human studies that examined the association of TRAF2 (1 study), TRAF4 (3 studies), and TRAF6 (3 studies) with survival rate in breast cancer patients (719 patients). As shown in Table 4, the present meta-analysis included 5 genetic association studies (TRAF4: 2 studies, 46 patients, and TRAF6: 2 studies, 212 patients).

**Table 4:** Summary of meta-analysis of included studies showing significant association of TRAF6 modulation and breast cancer survival rate.

Intervention	Outcome	Type of survival (no. studies)	Subgroup hazard ratio (95% CI)	Overall hazard ratio (95% CI)	Statistical method	Test for heterogeneity	Test for overall effect
TRAF6 expression	Cumulative survival rate	Kaplan–Meier survival analysis (2)	NA	1.01 [1.01, 1.01]	Hazard Ratio (IV, Fixed, 95% CI)	Chi^2^ = 0.06, df = 1 (P = 0.81); I^2^ = 0%	Z = 5.01 (P < 0.00001)

#### TRAF6 expression is associated with survival rate in breast cancer patients

Analysis of data from 212 patients collected from 2 pooled studies showed that high expression of TRAF6, not TRAF4, is associated with poor survival rate in breast cancer patients over a 5-year period (Log Hazard Ratio [HR]:1.01, 95% CI: 1.01, 1.01, P < 0.00001) (Table 4) (Table S8). A review of non-pooled studies (Table 5) confirmed that high expression of TRAF6 was detected in patient biopsies from both human breast carcinoma and lymph node metastasis^53^. Based on these findings from pooled studies it is reasonable to conclude that therapeutic targeting of TRAF6, but not TRAF2 and 4, can be of value in the treatment of breast cancer. However, evidence from non-pooled studies (Table 5) implicates both TRAF2^54^ and TRAF4^18,55–57^ in advanced breast cancer. Briefly, TRAF4 was found to be highly expressed in breast tumours^55,56^ and its expression is associated with disease- and relapse-free survival in breast cancer patients^18,57^. Similarly, TRAF2 expression was also found to be associated with distant metastasis-free survival in breast cancer patients^54^.

**Table 5:** Articles included in the narrative synthesis.

Study	Author/year	Title
In vitro	Mestre-Farrera et al. 2021 ^49^	Glutamine-directed migration of cancer-activated fibroblasts facilitates epithelial tumor invasion
	Wang et al. 2005 ^66^	Differential effect of anti-apoptotic genes Bcl-xL and c-FLIP on sensitivity of MCF-7 breast cancer cells to paclitaxel and docetaxel
	Kim et al. 2020^48^*	AMPK alpha 1 regulates lung and breast cancer progression by regulating TLR4-mediated TRAF6-BECN1 signaling Axis
	Sirinian et al. 2018^50^	RANK-c attenuates aggressive properties of ER-negative breast cancer by inhibiting NF-kappa B activation and EGFR signaling
	Liu et al. 2015^51^^+^	FOXP3 controls an miR-146/NF-kappa B negative feedback loop that inhibits apoptosis in breast cancer cells
	Liu et al. 2020^66^^+^	The MyD88 inhibitor TJ-M2010-2 suppresses proliferation, migration and invasion of breast cancer cells by regulating MyD88/GSK-3 beta and MyD88/NF-kappa B signalling pathways
	Zheng et al. 2015^45^^+^	CXCR4 3'UTR functions as a ceRNA in promoting metastasis, proliferation and survival of MCF-7 cells by regulating miR-146a activity
In vivo	Liu et al. 2017^20^^#^	Osteoclastic miR-214 targets TRAF3 to contribute to osteolytic bone metastasis of breast cancer
Human	Camilleri et al. 2007^56^*$	TRAF4 overexpression is a common characteristic of human carcinomas
	Regnier et al. 1995^53^	Presence of a new conserved domain in CART1, a novel member of the tumor necrosis factor receptor-associated protein family, which is expressed in breast carcinoma
	Wang et al. 2015^55^#	Expression of tumor necrosis factor receptor-associated factor 4 correlates with expression of Girdin and promotes nuclear translocation of Girdin in breast cancer
	Choi et al. 2013^54^^+^	EI24 regulates epithelial-to-mesenchymal transition and tumor progression by suppressing TRAF2-mediated NF-κB activity
	Zhou et al. 2014^57^^+^	TRAF4 mediates activation of TGF-β signaling and is a biomarker for oncogenesis in breast cancer
	Zhang et al. 2013^18^	TRAF4 promotes TGF-beta receptor signaling and drives breast cancer metastasis

^+^Data extracted but not pooled in meta-analysis; *data not extracted; ^$^in vivo data analysed by narrative synthesis. ^#^Human data analysed by narrative synthesis.

### Bioinformatics validation of meta-analysis outcomes

#### TRAF2/4/6 association with metastatic breast cancer in patients

**Figure 2:**
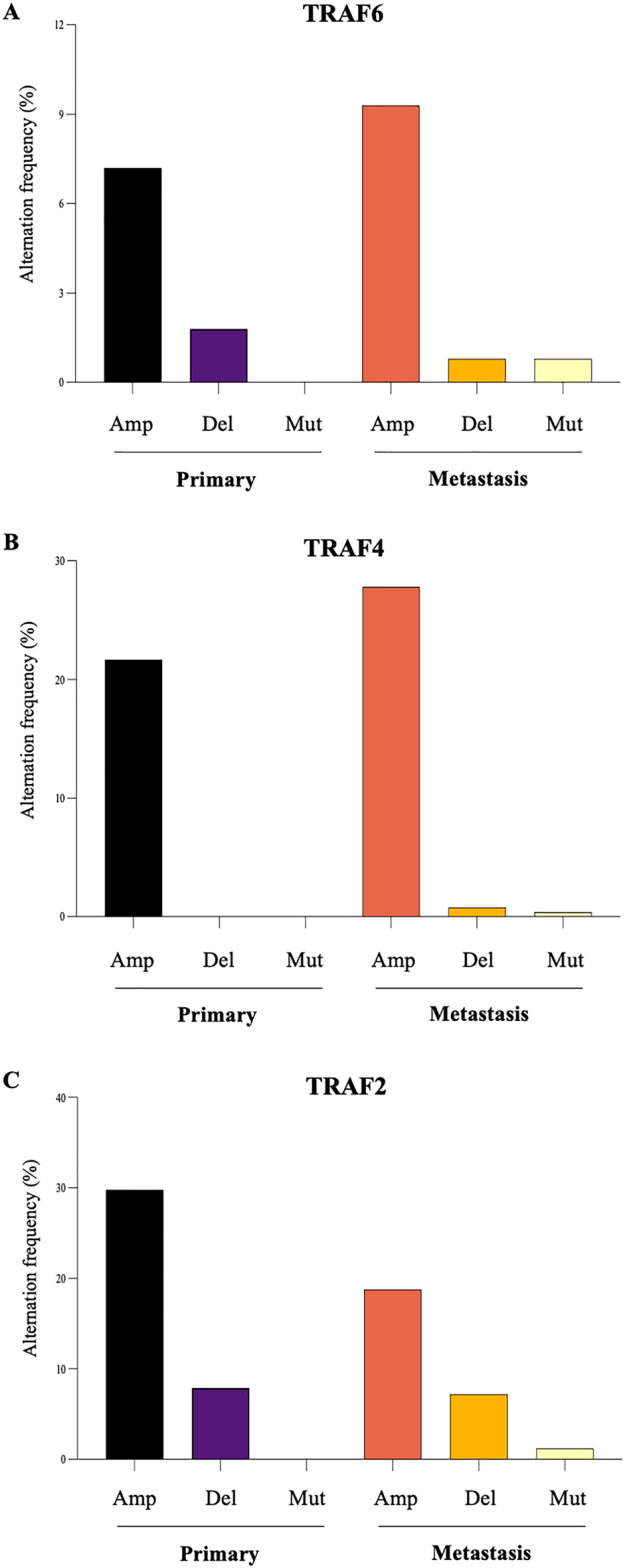
Genetic alterations in TRAFs in primary and metastatic breast cancer. Analysis of TRAF2 (**A**), TRAF4 (**B**) and TRAF6 (**C**) amplification (Amp), deletion (Del) and mutation (Mut) in tumours from patients of primary (n = 166) and metastatic (n = 335) breast cancer from the Metastatic Breast Cancer Project reported in the cBioPortal (https://www.cbioportal.org).

**Figure 3:**
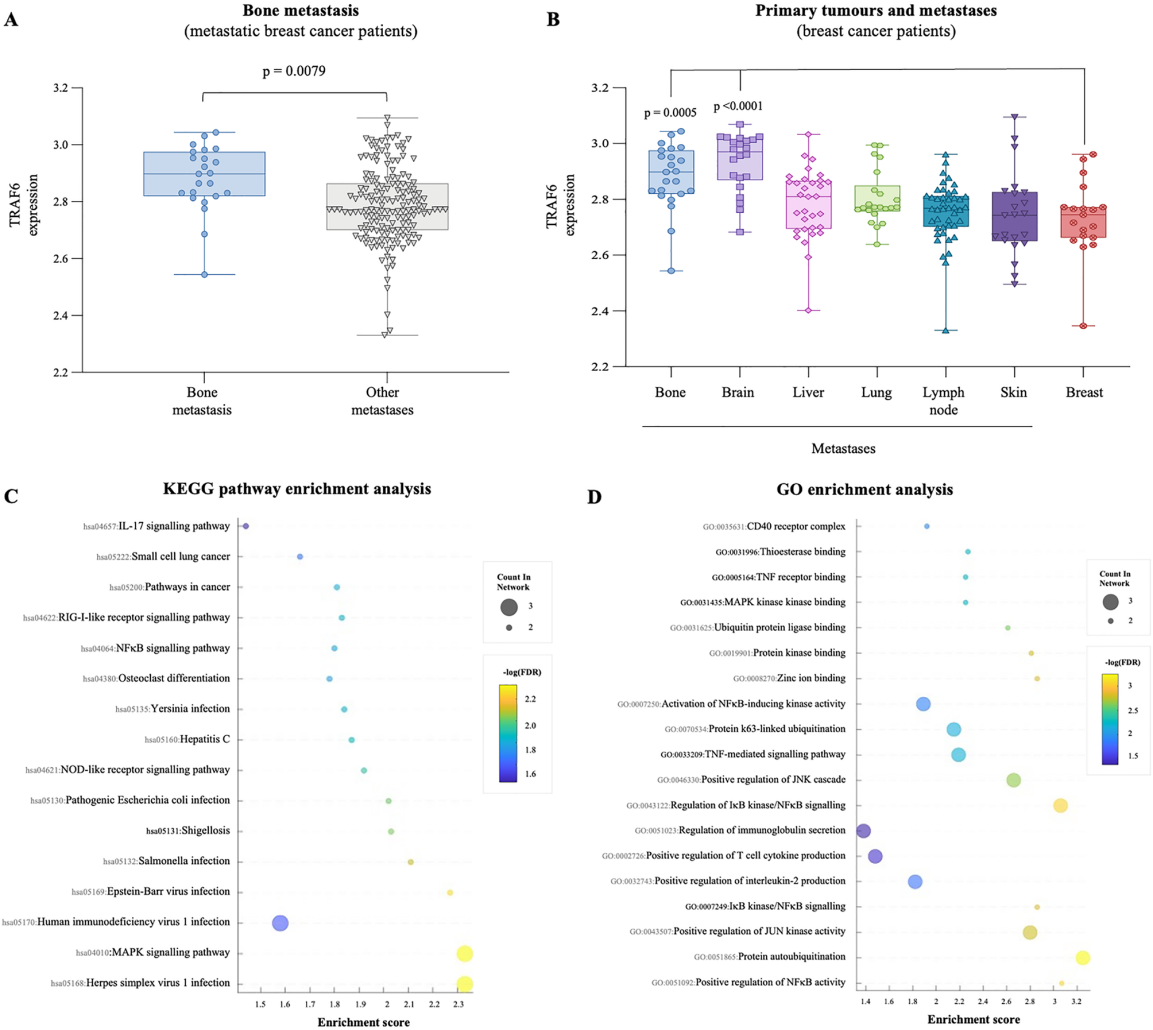
Differential expression and enrichment analysis of TRAFs. (**A**) TRAF6 expression in bone (n = 23) and other tumours (n = 161) from breast cancer patients. (**B**) TRAF6 expression in primary breast (n = 19), bone (n = 23), brain (n = 22), liver (n = 32), lung (n = 22), lymph nodes (n = 44), and skin (n = 22) tumours from breast cancer patients (The Molecular Taxonomy of Breast Cancer International Consortium (METABRIC) dataset)^64,65^. (**C**, **D)** KEGG pathway (**C**) and GO (**D**) enrichment analyses of TRAF2, 4, 6. The bubble charts ordinates show the path names, abscissa represents enrichment score, size of circles indicates the number of matching, and circle color represents the value of − log10 (FDR). FDR denotes False Discovery Rate. Permission has been obtained from Kanehisa laboratories for using KEGG pathway database (www.kegg.jp/kegg/kegg1.html).

**Figure 4:**
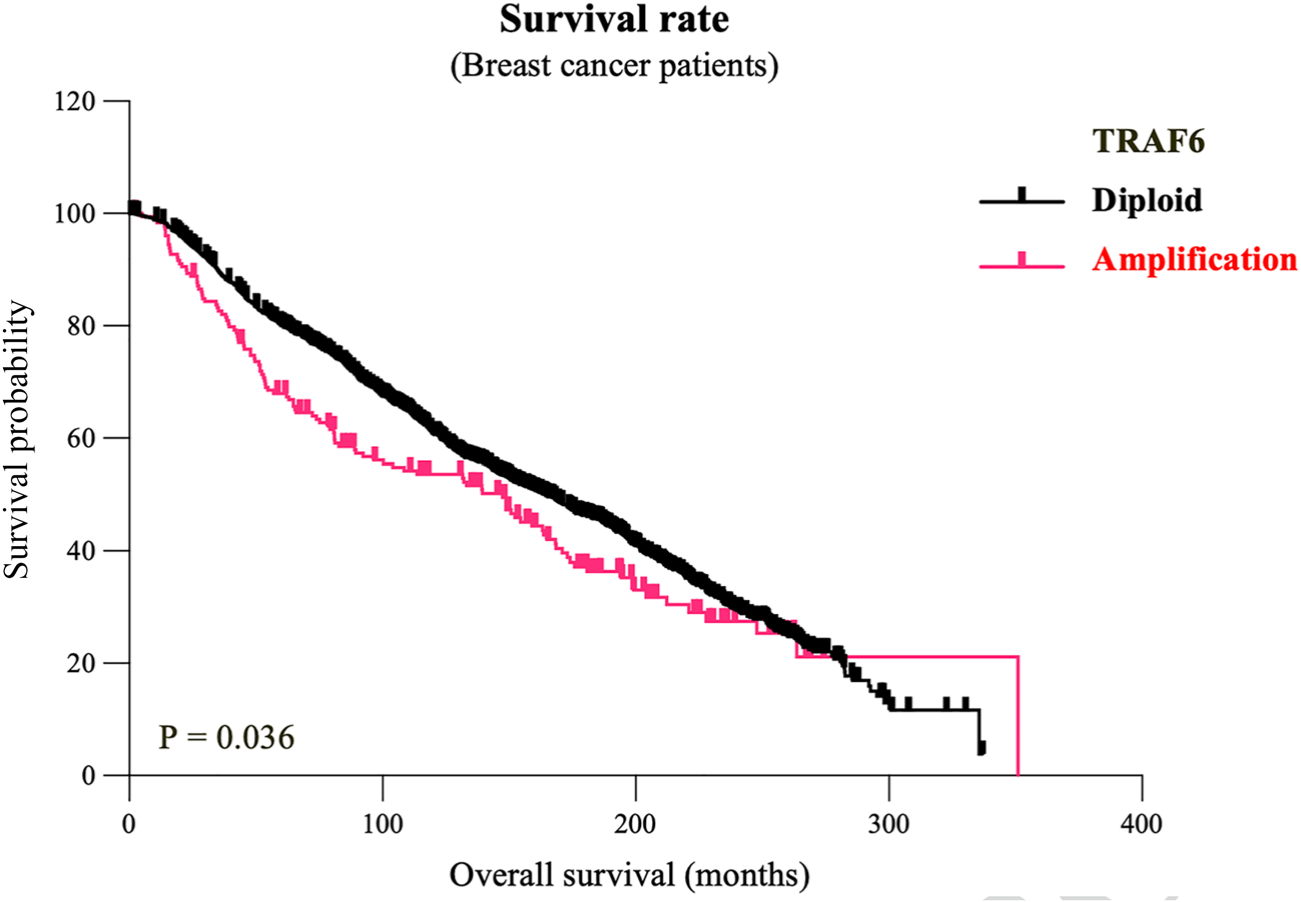
Genetic alterations in TRAF6 are associated with breast cancer survival. Analysis of copy number variations (CNVs) and cumulative survival rate in breast cancer patients (The METABRIC dataset). Patients are separated into those who were diploid for TRAF6 (n = 1738) and others who exhibited gain or amplification of TRAF6 (n = 213) (P = 0.036).

To further explore the role of TRAF2/4/6 in breast cancer, we went on to carry out a retrospective bioinformatics analysis (Figs. 2, 3 and 4), and Tables S12, S13). As shown in Fig. 2 (panels A and B), our investigation shows that TRAF6 as well as TRAF4 genes are highly amplified in samples from metastatic breast cancer patients (TRAF6: 9.3% and TRAF4: 27.8%) when compared primary tumours (TRAF6: 7.2% and TRAF4: 21.7%). In stark contrast, the TRAF2 gene is highly amplified in samples from primary tumours (29%) compared to 18.8% in those from metastatic breast cancer patients (Fig. 2C).

#### TRAF6 expression is associated with bone and brain metastasis

Further validation of clinical data using a large study population with 183 metastatic breast cancer patients was used to examine the association between the expression of TRAF2/4/6 and tissue-specific metastatic potential of advanced breast cancer (Fig. 3). As shown in Fig. 3 (panel A), the expression of TRAF6, but not TRAF2 and TRAF4, in patients with bone metastasis (n = 23) is significantly higher (p = 0.0079) than those with other metastases (n = 160). Further differential analysis of the data from this cohort revealed that TRAF6 expression in both brain (p = 0.0001) and bone metastases are significantly higher than that in primary breast tumours (Fig. 3, panel B).

#### KEGG pathway enrichment analysis for TRAF2/4/6

To gain more insight into the mechanism(s) by which TRAF6, as well as TRAF2 and 4, influence the progression and metastasis of breast cancer, we conducted a KEGG pathway enrichment analysis. As shown in Fig. 3 (panel C), a total of 16 pathways were significantly enriched (Fig. 3C, and Table S11). TRAF6 and TRAF2 (but no TRAF4) are enriched in differentiation of bone-resorbing osteoclasts (Fig. 4 and Table S11). Furthermore, TRAF6 and TRAF2 have also been found to be enriched in an additional 15 pro-inflammatory and immuno-modulatory pathways and processes implicated in breast cancer tumorigenesis and metastasis (Fig. 3C and Table S11). The list includes NFκB, Nod-like receptor, and MAPK signalling pathways, and pro-inflammatory and immuno-modulatory mediators such as retinoic acid-inducible gene I (RIG-I) and IL-17 (Fig. 3C and Table S11). In contrast, TRAF4 is only enriched in 3 pathways, namely pathways in cancer, small cell lung cancer, and IL-17 signaling pathway (Fig. 4 and Table S11).

#### GO enrichment analysis for TRAF2/4/6

Next, we utilized gene Ontology (GO) enrichment analysis to show that TRAF6 and TRAF2 are enriched in 19 processes and functions associated with NFκB and MAPK signal transduction, ubiquitination, and receptors for pro-inflammatory and immuno-modulatory factors such as CD40 and TNF (Fig. 3D and Table S12). In contrast, TRAF4 is only enriched in 50% of these terms (Fig. 3D and Table S12).

#### TRAF6 expression is associated with breast cancer survival

Next, we provide further evidence from bioinformatics analysis that confirms the findings from the aforementioned meta-analysis that uncovered the association of TRAF6 with survival rates in breast cancer patients. As shown in Fig. 4, breast cancer patients with gain or amplification of TRAF6 (n = 213) exhibited significantly lower overall survival rate compared to patients that are diploid (n = 1738) (p < 0.05).

## Discussion

### Study rationale and design

Metastasis is a major cause of death among women diagnosed with advanced breast cancer^58,59^. Thus, there is an urgent need to develop drugs that target novel key mechanism(s) involved in the regulation of metastatic behaviour of breast cancer cells. Inflammation plays a key role in all aspects of metastatic breast cancer^1,2^, and different pharmacological, genetic and molecular biology approaches have been used to study the involvement of the pro-inflammatory TRAF/NFκB axis in breast cancer—healthy cell interactions^7^. To explore which TRAF(s) to target for metastatic breast cancer treatment, we performed the present systematic review, meta-analysis and bioinformatics validation. The main aim of this investigation was to test the hypothesis that pharmacological modulation of TRAF1-7 can be of value in the treatment of advanced breast cancer. Thus, we included and paid particular attention to studies that featured standard breast cancer related methods, parameters and outcomes in cell culture systems, animal models and human studies. To explore the cellular changes and abnormalities that reflect the behaviour of metastatic breast cancer cells, our search strategy included in vitro and in vivo studies that examined human and animal breast cancer cell migration, invasion, adherence, apoptosis and proliferation in culture, and growth and metastasis in animals. In human, we first used meta-analysis to establish the association between TRAF1-7 expression and breast cancer survival in patients. Breast cancer is known to be driven by genomic copy number variations (CNVs)^60^. With this in mind, we then utilized bioinformatics analysis to validate the association between TRAF expression with tissue-specific metastases, and survival rate in primary and metastatic breast cancer patients. Finally, we performed pathway, function, and process enrichment analysis to gain a better understanding of the mechanism(s) by which TRAFs regulates the behaviour of breast cancer cells.

### Summary of findings

The meta-analysis and bioinformatics validation yielded eight key outcomes: (1) TRAF2/4/6 inhibition is associated with reduced breast cancer cell motility in vitro and tumour weight/volume in vivo; (2) TRAF2/4 inhibition is associated with reduced breast cancer cell adherence in vitro; (3) TRAF6 inhibition is associated with reduced breast cancer cell proliferation in vitro; (4) TRAF4/6 inhibition is associated with reduced breast cancer cell metastasis in vivo; (5) TRAF6 expression is associated with breast cancer survival rate; (6) TRAF6 expression is significantly higher in patients with secondary breast cancer in bone and brain; (7) TRAF6 and TRAF2 are involved in the regulation of osteoclast differentiation, and (8) TRAF2/4/6 are predominately involved in the regulation of pro-inflammatory and immuno-modulatory pathways and processes implicated in breast cancer metastasis. Overall, we tentatively conclude that TRAF6 is associated with breast cancer cell behaviour in vitro, tumour burden and metastasis in mice, and bone metastasis and survival rate in breast cancer patients.

### Therapeutic implications

TRAF6 is the most studied member of the TRAF family, and a number of studies have demonstrated its involvement in breast cancer, inflammation and immunity^1–7,15^. The findings from included human studies complement previous studies, but most importantly confirm the association between TRAF6 expression with poor survival rate and bone metastasis, two key features of advanced, metastatic breast cancer. This has important implications. First, it implies that TRAF6 may be a novel biomarker for the identification of metastatic breast cancer in patients, particularly those who are likely to benefit from combinational treatments such as anti-inflammatory agents, or even TRAF inhibitors. Secondly, present evidence adds weight and credence to the overall interpretation of in vitro, in vivo and human data that confirms the role of TRAF6/NFκB axis in the regulation of the ability of metastatic breast cancer cells to metastasise, grow in distant tissues, and influence the differentiation of healthy cells such as osteoclasts. Notwithstanding, evidence from meta-analysis and bioinformatics validations indicates that TRAF2 and TRAF4 are highly expressed in primary and metastatic tumours^55,56^, and their elevated level of expression is associated with metastasis and survival rate in breast cancer patients^54,57^. These are important observations since underlying mechanisms by which TRAF2, 4 and 6 individually or cooperatively regulate breast cancer cell behaviour are poorly understood and remain unexplored. In a recent pharmacological study from our laboratories, we showed that administration of a small-molecule that selectively inhibits TRAF6 activity by binding to the CD40 pocket in the TRAF6 protein was sufficient to reduce overt metastasis. However, to our surprise, treatment with this compound failed to reduce breast cancer-induced bone loss, a hallmark of advanced breast cancer in the skeleton^40^. Breast cancer cell growth, mobility and their interaction with bone and immune cells in the skeleton is regulated by a myriad of immune- and bone-derived factors. The list includes TNFα, IL1β and TGFβ that are known to act both dependently and independently of the TRAF6/CD40 axis. Thus, it is tempting to speculate that agents that target multiple TRAFs—particularly TRAF2/4/6—may represent a more promising strategy to treat a multi-factorial and -faceted disease such as metastatic breast cancer. However, future studies that explore this hypothesis should be mindful of potential off-target side-effects associated with such multi-targeted therapy^61^, such as manipulation of the immune response^3–5^, that may limit their usefulness. Thus, the therapeutic relevance of current evidence is limited. Furthermore, careful exploration of potential off/side effects associated with inhibition of multiple TRAFs in healthy cells in the tumour micro-environment, coupled with utilization of immuno-competent animal models will aid with our understanding of the therapeutic potential of TRAF inhibitors in breast cancer, and ultimately guide future clinical research in human.

### Strengths and limitations

The strength of our present meta-analysis is warranted by the systematic approach and comprehensive appraisal of up-to-date evidence on articles and featured studies in three major databases, namely Medline, Web of Science and Scopus, coupled by the use of the online tool WebPlotDigitizer (https://apps.automeris.io/wpd/) to obtain the mean and standard deviation or standard error measurement from relevant figures in all included in vitro, in vivo and human studies. Thus, no study data was deemed non-retrievable. Additionally, our investigation emphasises the importance of combining evidence from meta-analysis of in vitro, in vivo and human studies with bioinformatics validation to explore which TRAF represents a potential druggable target for breast cancer treatment. Furthermore, the evidence from in vitro and in vivo studies is supported by a wide range of experimental techniques and strategies that examined TRAF modulation using pharmacological, molecular biology and genetic approaches. Using explicit criteria for metastatic behaviour of breast cancer cells in vitro and in vivo together with metastasis and survival rate in breast cancer patients ensured a degree of homogeneity of the study outcomes. Admittedly, there were several limitations in our study: (1) the number of relevant articles included in the meta-analysis and bioinformatics validation is low; (2) a number of included articles featured more than one study type (i.e. in vitro, in vivo and/or human study); (3) the search was restricted to English language articles; (4) included in vivo studies were restricted to mouse experiments that used xenograft models, immortalized breast cancer cell lines and specific mouse strains^62,63^; (5) different non-selective TRAF inhibitors, concentrations/doses, administration schemes, treatment regimes, and analytical techniques used are likely to influence the reported outcomes of in vitro and in vivo studies; (6) the reported breast cancer survival rate is likely to be affected by diversity among patients studied; (7) the small-study effect cannot be excluded because of the insufficient number of relevant, pooled studies needed to perform subgroup analyses, meta-regression, Egger’s test and/or Funnel plot analysis. Thus, the clinical relevance of current evidence is limited; (8) more studies with a large study population are needed to confirm the role of TRAF6 in secondary breast cancer in the skeleton.

## Conclusion

Breast cancer metastasis continues to be a cause of death in patients. Our meta-analysis of pooled in vitro, in vivo*,* and human studies, coupled together with bioinformatics validation confirm that TRAF6 inhibition may be of value in the management of multiple aspects of advanced breast cancer. However, evidence from included studies indicates that TRAF2 and TRAF4 are also implicated in metastatic breast cancer. Thus, we hypothesise that agents that target multiple TRAFs, particularly TRAF2/4/6, can be of greater therapeutic value in the treatment of difficult-to-treat breast cancer subtypes. However, we caution that potential side-effects associated with multi-TRAF inhibition remain unclear. Furthermore, low study number coupled with heterogenicity of in vitro and in vivo models on which the aforementioned hypothesis is based, limit the translation of findings into clinical practice. Thus, further preclinical validation of the anti-tumour, anti-metastatic and anti-osteolytic efficacy of TRAF inhibitors in multiple models of advanced breast cancer that recapitulate the progression of metastatic breast cancer in humans is warranted, and ongoing.

## Supplementary Information

Supplementary Information.

## Data Availability

The datasets used and analysed in the present study are available from the public sources described.
