# Supplementary material for: TRAF6 as a potential target in advanced breast cancer: a systematic review, meta-analysis, and bioinformatics validation
Source: Sci Rep. 2023 Mar 21;13:4646. doi: 10.1038/s41598-023-31557-0 (PMC10029787; doi:10.1038/s41598-023-31557-0)
Supplement: Supplementary file 1 — Supplementary Information. [file 41598_2023_31557_MOESM1_ESM.docx]

*Zeng et. al*. TRAF6 as a potential target in advanced breast cancer: a systematic review, meta-analysis, and bioinformatics validation

**SUPPLEMENTARY MATERIALS**

**Content**

**1. Supplementary tables**

**Table S1.** Search strings for Medline, Web of Science and Scopus. **2**

**Table S2.** List of excluded studies **3**

**Table S3.** Characteristics and outcomes of *in vitro* studies **4**

**Table S4.** Characteristics and outcomes of *in vivo* studies **11**

**Table S5.** Characteristics and outcomes of breast cancer patient studies **14**

**Table S6.** Summary of meta-analysis showing non-significant association of cell behaviours changes

in in vitro breast cancer cell lines with pharmacological and genetic modulation of TRAF 2/4/6 **16**

**Table S7.** Summary of meta-analysis showing non-significant association of tumour weight/ volume

and overt metastasis in *in vivo* model with pharmacological and genetic modulation of TRAF 2/4/6 **17**

**Table S8.** Summary of meta-analysis showing non-significant association between TRAF4 expression

and breast cancer patient survival **18**

**Table S9.** Quality assessment for human studies **19**

**Table S10.** PRISMA checklist for article. **20**

**Table S11**. PRISMA checklist for abstract. **23**

**Table S12.** KEGG enrichment analysis of TRAF2, 4, 6. **24**

**Table S13.** GO enrichment of TRAF2, 4, 6. **25**

**2. Supplementary figures**

Figure S 1. Risk of bias (RoB) assessment for *in vivo* studies (SYRCLE RoB tool.) **26**

Figure S 2. Risk of bias (RoB) assessment for in vitro studies (OHAT RoB tool.) **27**

**3. Supplementary reference 26**

1. **Supplementary tables**

**Table S1.** Search strings for Medline, Web of Science and Scopus.

| **PubMed (Medline)** | **Web of Science** | **Scopus** |
| --- | --- | --- |
| 1. ('cancer' or '*carcinoma' or 'neoplasm$' or 'tumo?r).tw. 2. "tumor necrosis factor receptor-associated peptides and proteins"/ 3. 'TRAF*'.tw. 4. ('TRAF* adj3 protein$').tw. 5. ('TNF* receptor$ associated factor$ family' or 'TRAF* family').tw. 6. ('TNF* receptor$ associated factor$' or 'TNF* receptor$-associated factor$').tw. 7. ('Tumo?r necrosis factor$ receptor$ associated factor$' or 'Tumo?r necrosis factor$ receptor$-associated factor$').tw. 8. 'TRAF$ interacti* protein$'.tw. 9. 'Adaptor protein*'.mp. 10. 'E3 ligase$'.mp. 11. ('Breast Neoplasm$' or 'Neoplasm$, Breast').tw. 12. ('Breast tumo?r' or 'tumo?r, breast').tw. 13. ('breast cancer$' or 'cancer$, breast').tw. 14. ('Mammary Cancer$' or 'Cancer$, Mammary').tw. 15. ('Breast Malignant Neoplasm$' or 'Malignant Neoplasm$ of Breast').tw. 16. ('*Mammary Carcinoma$' or 'Carcinoma$, * Mammary').tw. 17. ('*Mammary Neoplasm$' or 'Neoplasm$, * Mammary').tw. 18. ('*Breast Carcinoma$' or 'Carcinoma$, * Breast').tw. 19. ('Breast metastas*s' or 'metastas*s, Breast').tw. 20. ('Breast adj2 metastas*s').tw. 21. ('Mammary adj2 metastas*s').tw. 22. review.pt. 23. 10 or 9 or 8 or 7 or 6 or 5 or 4 or 3 or 2 24. 18 or 17 or 16 or 15 or 14 or 13 or 12 or 11 25. 21 or 20 or 19 26. 1 and 23 and 24 and 25 27. 26 not 22 | 1. TS=('cancer' or '*carcinoma or neoplasm$' or 'tumo?r’) 2. TS=('TRAF*’) 3. TS=('TRAF*’ protein$’) 4. TS=('TNF* receptor$ associated factor$ family' or 'TRAF* family’) 5. TS=('TNF* receptor$ associated factor$' or 'TNF* receptor$-associated factor$’) 6. TS=('Tumo?r necrosis factor$ receptor$ associated factor$' or 'Tumo?r necrosis factor$ receptor$-associated factor$’) 7. TS=('TRAF$ interacti* protein$’) 8. TS=('Adaptor protein*’) 9. TS=('E3 ligase$’) 10. TS=('Breast Neoplasm$' OR 'Neoplasm$, Breast’) 11. TS=('Breast tumo?r' OR 'tumo?r, breast’) 12. TS=('breast cancer$' OR 'cancer$, breast’) 13. TS=('Mammary Cancer$' OR 'Cancer$, Mammary’) 14. TS=('Breast Malignant Neoplasm$' OR 'Malignant Neoplasm$ of Breast’) 15. TS=('*Mammary Carcinoma$' OR 'Carcinoma$, *Mammary’) 16. TS=('*Mammary Neoplasm$' or ‘Neoplasm$, *Mammary’) 17. TS=('*Breast Carcinoma$' OR 'Carcinoma$, *Breast’) 18. TS=('Breast metastas*s' OR 'metastas*s , Breast’) 19. #17 OR #16 OR #15 OR #14 OR #13 OR #12 OR #11 OR #10 20. #9 OR #8 OR #7 OR #6 OR #5 OR #4 OR #3 OR #2 21. #20 AND #18 AND #19 AND #1 22. (#21)  AND DOCUMENT  TYPES: (Review) 23. #21 NOT #22 | 1. TITLE-ABS-KEY ( cancer  OR  *carcinoma  OR  neoplasm$  OR  tumo?r ) 2. TITLE-ABS-KEY ( "tumor necrosis factor receptor-associated peptides and proteins" ) 3. TITLE-ABS-KEY ( traf* ) 4. TITLE-ABS-KEY ( "TRAF* protein$" ) 5. TITLE-ABS-KEY ( "TNF* receptor$ associated factor$ family"  OR  "TRAF* family" ) 6. TITLE-ABS-KEY ( "TNF* receptor$ associated factor$"  OR  "TNF* receptor$-associated factor$" ) 7. TITLE-ABS-KEY ( "Tumo?r necrosis factor$ receptor$ associated factor$"  OR  "Tumo?r necrosis factor$ receptor$-associated factor$" ) 8. TITLE-ABS-KEY ( "TRAF$ interacti* protein$" ) 9. TITLE-ABS-KEY ( "Adaptor protein*" ) 10. TITLE-ABS-KEY ( "E3 ligase$" ) 11. TITLE-ABS-KEY ( "Breast Neoplasm$"  OR  "Breast tumo?r"  OR  "breast cancer$"  OR  "Mammary Cancer$"  OR  "Breast Malignant Neoplasm$"  OR  "*Mammary Carcinoma$"  OR  "*Mammary Neoplasm$"  OR  "*Breast Carcinoma$" ) 12. TITLE-ABS-KEY ( "metastas*s" ) 13. #10 or #9 or #8 or #7 or #6 or #5 or #4 or #3 or #2 14. #13 and #12 and #1 |

**Table S2.** List of excluded studies

| **Studies** | **Reason for exclusion** |
| --- | --- |
| Brincas *et al*., 2019 ^1^ | No relevant data. |
| Billir *et al*., 2015 ^2^ | No relevant data. |
| Chan *et al*., 2012 ^3^ | No relevant data. |
| He *et al*., 2020 ^4^ | No relevant data. |
| Helbig *et al*., 2003 ^5^ | No relevant data. |
| Li *et al*., 2010 ^6^ | Review. |
| Li *et al*., 2020 ^7^ | No relevant data. |
| Moelans *et al*., 2014 ^8^ | No relevant data. |
| Niu *et al*., 2021 ^9^ | No relevant data. |
| Patel *et al*., 2000 ^10^ | No relevant data. |
| Qi *et al*., 2021 ^11^ | No relevant data. |
| Sato *et al*., 2020 ^12^ | No relevant data. |
| Song *et al*., 2015 ^13^ | Book chapter. |
| Sun *et al*., 2019 ^14^ | No relevant data. |
| Tomasetto *et al*., 1998 ^15^ | Review. |
| Van *et al*., 2020 ^16^ | No relevant data. |

**Table S3.** Characteristics and outcomes of in vitro studies

| *Study* | *Species* | *Intervention (pharmacological or genetic manipulation)* | *Target* | *Method of analysing study outcomes* | *Funding source* |
| --- | --- | --- | --- | --- | --- |
| Bishop *et al*. 2020 ^17^ | Human | 6877002 | Inhibit TRAF6 | Migration and invasion of MDA-231-BT cells. | Cancer Research UK Development Fund (University of Edinburgh) and funding from Breast Cancer Now (University of Sheffield). |
| Choi *et al*. 2013 ^18^ | Mouse | Ei24 shRNA and overexpression | Upregulate and inhibit TRAF2 | Migration of B16F10 cells. | National Research Foundation of Korea (NRF) funded by the MEST (2009-0081177 and 2010-0020878), National R&D Program for Cancer Control, MoHWFA (1020220), and Bio-industry Technology Development Program, MAFRA (311054-03-2-HD110), Republic of Korea. |
| Jang *et al*. 2011 ^19^ | Human | TRAF2-siRNA | Inhibit TRAF2 | Migration of MDA-MB-231 cells. | Basic Science Research Program through the National Research Foundation of Korea (NRF) funded by the Ministry of Education, Science and Technology (grant no. 2009-0075206 & grant no. 2011-0003981). This study was also supported by a faculty research grant of Yonsei University College of Medicine for 2010 (8-2010-0023). |
| Jiang *et al*. 2016 ^20^ | Human | miRZip-892b | Upregulate TRAF2 | Invasion and colony formation of MDA-MB-231 and ZR-75-30 cells. | Ministry of Science and Technology of China grant (973 Program, no. 2014CB910604); the Distinguished Young Scholar of Guangdong Province, China (no. 2015A030306033);GDUPS (2012); Natural Science Foundation of China (81325013, 81530082, 81201548, 81201546, and 91529301); the Science and Technology of Guangdong Province (No. 2013B021800096, 2015A030313468, 2014A030313008, and 2014A030313220); the Guangdong special Support program (2014TX01R076); the Guangzhou scholars research projects of Guangzhou municipal colleges and universities (no. 12A009D); Pearl River projects (Young Talents of Science and Technology) in Guangzhou (no. 2013J2200028); and Foundation of Key Laboratory of Gene Engineering of the Ministry of Education. |
| Li *et al*. 2012 ^21^ | Human | Plumbagin | Inhibit TRAF6 | Migration, invasion and viability of MDA-MB-231 and BT549 cells. | 973 Program (2012CB910400, 2010CB529704), National Natural Science Foundation of China (30800653, 30930055 and 81071437) and the Fundamental Research Funds for the Central Universities. |
| Liu *et al*. 2015 ^22^ | Human | miRNA-146a/b inhibitor | Upregulate TRAF6 | Migration of MCF7 cells. | NIH/National Cancer Institute (CA164688, CA179282, and CA118948; L. Wang), the Department of Defense (PC130594; L. Wang and W.-H. Yang), the UAB Faculty Development Grant (R. Liu), the Larsen Endowment Fellowship Program Grant (W.-H. Yang), and the Mercer University Seed Grant (W.-H. Yang). |
| Liu *et al*. 2020 ^23^ | Human | TJ-M2010-2 | Inhibit TRAF6 | Migration, invasion, proliferation and cell apoptosis of MCF7 and MDA-MB-231 cells. | National Natural Science Foundation of China (Grant No. [81802895](https://www.sciencedirect.com/science/article/pii/S0014482720304043?via%3Dihub#gs1)), the Health and Family Planning Commission of Wuhan Municipality (Grant No. [WX17Q16](https://www.sciencedirect.com/science/article/pii/S0014482720304043?via%3Dihub#gs2)), the Fundamental Research Funds for the Central Universities (Grant No. [2042019kf0229](https://www.sciencedirect.com/science/article/pii/S0014482720304043?via%3Dihub#gs3)). |
| Peramuhendige *et al*. 2018 ^24^ | Human | TRAF2 overexpression and knockdown | Upregulate and inhibit TRAF2 | Migration (% wound closure) and invasion (% distance) of MDA-MB-231 cells. | Breast Cancer Now, Darwin Endowment Fund and Cancer Research UK. |
| Shi *et al*. 2019 ^25^ | Mouse | TLR5 overexpression | Upregulate TRAF6 | Proliferation of 4T1 cell. | National Natural Science Foundation of China (81371601, to G.H.) and the Natural Science Foundation of Shandong Province (ZR2019MH019, to G.H.) |
| Wang *et al*. 2013 ^26^ | Human | TRAF4-siRNA,  TRAF4-overexpression | Inhibit TRAF4,  Upregulate TRAF4 | Migration of MCF7 cells, proliferation of MCF7 cells. | Not stated. |
| Wang *et al*. 2021 ^27^ | Human | miRNA-7 | Inhibit TRAF6 | Migration of MCF7 cell. | Youth High End Talent Cultivation Project of Peihua University. |
| Yao *et al*. 2017 ^28^ | Human | Wogonoside | Inhibit TRAF2/4 | Migration, invasion, adhesion of MDA-MB-231, MDA-MB-435, BT-474 cells. | National Science & Technology Major Project (No. 2017ZX09301014, 2017ZX09101003-005-023, 2017ZX09101003-003-007), Program for Changjiang Scholars and Innovative Research Team in University  (IRT1193), the Project Program of State Key Laboratory of Natural Medicines, China Pharmaceutical University (SKLNMZZCX201606), the National Natural Science Foundation of China (No. 81603135, 81673461, 81373449, 81373448), the Fundamental Research Funds for the Central Universities (2016ZPY005). |
| Zhang *et al*. 2013 ^29^ | Human | TRAF4-shRNA | Inhibit TRAF4 | Migration (trans-well cell numbers), invasion of MDA-MB-231 cells. | A Netherlands Organization of Scientific Research grant (MW-NWO 918.66.606), Cancer Genomics Centre Netherlands, and the Centre for Biomedical Genetics. This work was supported in part by Key Construction Program of the National ‘‘985’’ Project and Zhejiang University Special Fund for Fundamental Research, as well as the Fundamental Research Funds for the Central Universities. |
| Zheng *et al*. 2015 ^30^ | Human | miRNA-146a mimics/inhibitor | Inhibit/upregulate TRAF6 | Migration, invasion, proliferation and adhesion of MCF7 cells. | National Natural Science Foundation of China [Grants [81372331](https://www.sciencedirect.com/science/article/pii/S0171933515000564?via%3Dihub#gs1)] (to Tao Xi), Major Drug Discovery of Science and Technology Major Projects [Grants [2009ZX09103-652](https://www.sciencedirect.com/science/article/pii/S0171933515000564?via%3Dihub#gs2)] and the project funded by the Priority Academic Program Development (PAPD) of Jiangsu Higher Education Institutions. |

**Table S4. Characteristics and outcomes of in vivo studies**

| *Study* | *Species* | *Intervention (pharmacological or genetic manipulation)* | *Target* | *Methods of analysing study outcomes* | *Funding source* | |
| --- | --- | --- | --- | --- | --- | --- |
| Bishop *et al*. 2020 ^17^ | | Mouse | 6877002 | Inhibit TRAF6 | Bone metastasis (log_2_ photons/sec) of 4T1 intracardiac injection BALB/c mice (female, 8 weeks of age). | Cancer Research UK Development Fund (University of Edinburgh) and funding from Breast Cancer Now (University of Sheffield). |
| Jiang *et al*. 2016 ^20^ | | Mouse | miR-892b | Inhibit TRAF2 | Lung metastasis (surface metastasis nodules) of MDA-MB-231 cell xenograft BALB/c nude mice (female, 4-5 weeks of age, 18-20g). | Ministry of Science and Technology of China grant (973 Program, no. 2014CB910604); the Distinguished Young Scholar of Guangdong Province, China (no. 2015A030306033); GDUPS (2012); Natural Science Foundation of China (81325013, 81530082, 81201548, 81201546, and 91529301); the Science and Technology of Guangdong Province (No. 2013B021800096, 2015A030313468, 2014A030313008, and 2014A030313220); the Guangdong special Support program (2014TX01R076); the Guangzhou scholars research projects of Guangzhou municipal colleges and universities (no. 12A009D); Pearl River projects (Young Talents of Science and Technology) in Guangzhou (no. 2013J2200028); and Foundation of Key Laboratory of Gene Engineering of the Ministry of Education. |
| Lin *et al*. 2014 ^31^ | | Mouse | TRAF6-shRNA | Inhibit TRAF6 | Lung metastasis (numbers of lung metastases nodules) of MDA-MB-231 cell intracardiac injection nude mice (female, 6 weeks of age). | The National Basic Research Program (2011CB510106), the National Natural Science Foundation of China (30971137, 31171308, and 81172208), the National High Technology  Research and Development Program of China (2013AA032201), and the Science and Technology Commission of Shanghai Municipality (10140901600 and 11DZ1910200). |
| Liu *et al.* 2015 ^22^ | | Mouse | miRNA146a/b-inhibitors | Upregulate TRAF6 | Lung metastasis tumour burden (% tissue area) of MDA-MB-231 cell intravenously injected into 8-week-old female NSG mice. | NIH/National Cancer Institute (CA164688, CA179282, and CA118948; L. Wang), the Department of Defense (PC130594; L. Wang and W.-H. Yang), the UAB Faculty Development Grant (R. Liu), the Larsen Endowment Fellowship Program Grant (W.-H. Yang), and the Mercer University Seed Grant (W.-H. Yang). |
| Liu *et al*. 2020 ^23^ | | Mouse | TJ-M2010-2 | Inhibit TRAF6 | Tumour volume (mm^3^) of MDA-MD-231 and MCF7 cell xenograft BALB/c nude mice (female, 5 weeks of age). | National Natural Science Foundation of China (Grant No. [81802895](https://www.sciencedirect.com/science/article/pii/S0014482720304043?via%3Dihub#gs1)), the Health and Family Planning Commission of Wuhan Municipality (Grant No. [WX17Q16](https://www.sciencedirect.com/science/article/pii/S0014482720304043?via%3Dihub#gs2)), the Fundamental Research Funds for the Central Universities (Grant No. [2042019kf0229](https://www.sciencedirect.com/science/article/pii/S0014482720304043?via%3Dihub#gs3)). |
| Peramuhendige *et al*. 2018 ^24^ | | Mouse | TRAF2-overexpression | Upregulate TRAF2 | Tumour growth (% tissue area) of MDA-MB-231 cell orthotropic injection into mammary fat pads of adult mice. | Breast Cancer Now, Darwin Endowment Fund and Cancer Research UK. |
| Rezaeian *et al.* 2017 ^32^ | | Mouse | TRAF6-overexpression | Upregulate TRAF6 | Tumour volume (×100 mm3) of MDA-MB-231 cell 6 weeks after subcutaneously injection into the right flank of 6-week-old nude mice. | NIH R01 grants (R01CA182424- 01A1, R01CA193813-01), the MD Anderson Cancer Center SPORE development grant, the R. Clark Fellowship award, MD Anderson Cancer Center Prostate Moonshot Program funds, and Start-up funds from Wake Forest University School of Medicine to H.K.L. and MOST104-2314-B-384-009-MY3 and MOHW104-TDU-M-212-133004 grants from Taiwan to C.F,L. |
| Shi *et al.* 2019 ^25^ | | Mouse | TLR5 knockdown | Upregulate TRAF6 | Tumour volume (mm3) of 4T1 cell model mice 6 days after injection into the lower left and right flanks of male nude mice. | National Natural Science Foundation of China (81371601, to G.H.) and the Natural Science Foundation of Shandong Province (ZR2019MH019, to G.H.) |
| Yao *et al*. 2017 ^28^ | | Mouse | Wogonoside | Inhibit TRAF2/4 | Tumour volume (mm^3^) of MDA-MB-231 cell orthotopic BALB/c nude mice (female, 4 weeks of age). | National Science & Technology Major Project (No. 2017ZX09301014, 2017ZX09101003-005-023, 2017ZX09101003-003-007), Program for Changjiang Scholars and Innovative Research Team in University  (IRT1193), the Project Program of State Key Laboratory of Natural Medicines, China Pharmaceutical University (SKLNMZZCX201606), the National Natural Science Foundation of China (No. 81603135, 81673461, 81373449, 81373448), the Fundamental Research Funds for the Central Universities (2016ZPY005). |
| Zhang *et al*. 2013 ^29^ | | Mouse | TRAF4-shRNA | Inhibit TRAF4 | Bone metastasis (numbers of bone metastasis, bone metastasis BLI signals) of MDA-MB-231 cell intracardiac injection TRAF4^-/-^ mice. | A Netherlands Organization of Scientific Research grant (MW-NWO 918.66.606), Cancer Genomics Centre Netherlands, and the Centre for Biomedical Genetics. This work was supported in part by Key Construction Program of the National ‘‘985’’ Project and Zhejiang University Special Fund for Fundamental Research, as well as the Fundamental Research Funds for the Central Universities. |
| Zhu *et al*. 2018 ^33^ | | Mouse | TRAF4-shRNA | Inhibit TRAF4 | Tumour volume (mm^3^) of MDA-MB-231 cell orthotopic BALB/c nude mice (female, 4-6 weeks of age, 16-20g). | National Natural Science Foundation of China (no. [81260394](https://www.sciencedirect.com/science/article/pii/S0006291X18309513?via%3Dihub#gs1)). |

**Table S5. Characteristics and outcomes of breast cancer patient studies**

| *Study* | *Study center or data source* | *Study population* | *Outcome (Type of survival)* | *Target* | *Sample size (High expression/Low expression)* | *P Value* | *HR (95%CI)* | *Ln (HR), SE* | *Funding Source* |
| --- | --- | --- | --- | --- | --- | --- | --- | --- | --- |
| Lin et al. 2014 ^31^ | Shanghai Ruijin Hospital,  Shanghai Jiao Tong University, School of Medicine | Breast cancer patients | Overall survival | TRAF6 | 134 (33/101) | <0.01 | 2.26 (1.23-3.30) | 0.8154, 0.3104 | National Basic Research Program (2011CB510106), the National Natural Science Foundation of China (30971137, 31171308, and 81172208), the National High Technology Research and Development Program of China (2013AA032201), and the Science and Technology Commission of Shanghai Municipality (10140901600 and 11DZ1910200). |
| Rezaeian et al. 2017 ^32^ | Chi-Mei Foundational Medical Center | Taiwanese cohort of human breast carcinomas | Disease specific survival | TRAF6 | 212^#^ (NA/NA) | 0.017 | 1.008 (1.001-1.015) | 0.008, 0.0036 | NIH R01 grants (R01CA182424- 01A1, R01CA193813-01), the MD Anderson Cancer Center SPORE development grant, the R. Clark Fellowship award, MD Anderson Cancer Center Prostate Moonshot Program funds, and Start-up funds from Wake Forest University School of Medicine to H.K.L. and MOST104-2314-B-384-009-MY3 and MOHW104-TDU-M-212-133004 grants from Taiwan to C.F,L. |
| Rezaeian et al. 2017 ^32^ | Chi-Mei Foundational Medical Center | Taiwanese cohort of human breast carcinomas | Metastasis-free survival | TRAF6 | 212^#^ (NA/NA) | <0.001 | 1.009 (1.005-1.013) | 0.009, 0.002 |  |
| Zhang et al. 2013 ^29^ | NA | Breast cancer patients | Relapse free period for 20 years | TRAF4 | 327 (129/198) | 0.049 | 1.36 (1.001-1.845) | 0.307, 0.024 | Netherlands Organization of Scientific Research grant (MW-NWO918.66.606), Key Construction Program of the National ‘‘985’’ Project and Zhejiang University Special Fund for Fundamental Research and Fundamental Research Funds for  the Central Universities. |
| Zhao et al. 2015 ^34^ | Department of Pathology of the First Affiliated Hospital of China Medical University | Chinese breast cancer patients | Overall survival | Expression of TRAF2 in the Cytoplasm of Malignant Plural Effusion Cells of Breast Cancer | 46^$^ (34/12) | NA | 2.03 (0.96-4.29) ^*^ | 0.71, 0.38 | Natural Scientific Foundation of China (81572615). |
| Zhao et al. 2015 ^34^ | Department of Pathology of the First Affiliated Hospital of China Medical University | Chinese breast cancer patients | Overall survival | TRAF4 in cytoplasm | 46^$^ (40/6) | NA | 4.45 (1.10-18.06) ^*^ | 1.49, 0.76 | Natural Scientific Foundation of China (81572615). |
| Zhao et al. 2015 ^34^ | Department of Pathology of the First Affiliated Hospital of China Medical University | Chinese breast cancer patients | Overall survival | TRAF4 in nuclei | 46^$^ (20/26) | NA | 0.12 (0.03-0.33) ^*^ | -2.16, 0.54 | Natural Scientific Foundation of China (81572615). |

^*^Refers to HR (95%CI) are estimated as previously described in Tierney et al. 2007. ^$^ refers to studies using the same cohort of patients’ samples but patients were stratified by different signatures. ^#^ refers to studies using the same cohort of patients’ sample but evaluate different outcomes via univariate or multivariate survival analyses.

**Table S6.** Summary of meta-analysis showing non-significant association of cell behaviours changes in in vitro breast cancer cell lines with pharmacological and genetic modulation of TRAF 2/4/6

| **Outcome** | | **Intervention** | **Type of cell cultures (no. studies)** | **Overall (std.) mean difference (95% CI)** | **Statistical method** | **Test for heterogeneity** | **Test for overall effect** |
| --- | --- | --- | --- | --- | --- | --- | --- |
| **TRAF4** | **Proliferation** | Genetic inhibition | MCF7 (3) | -0.01 [-0.03, 0.02] | Mean Difference (IV, Fixed, 95% CI) | Chi² = 0.47, df = 2 (P = 0.79); I² = 0% | Z = 0.65 (P = 0.52) |
| **TRAF6** | **Migration** | Pharmacological inhibition | BT459 (3) | -5.60 [-11.76, 0.56] | Std. Mean Difference (IV, Random, 95% CI) | Tau² = 17.19; Chi² = 4.68, df = 2 (P = 0.10); I² = 57% | Z = 1.78 (P = 0.07) |
|  |  |  | MCF7 (1) | -9.20 [-18.23, -0.17] | Std. Mean Difference (IV, Fixed, 95% CI) | *NA* | Z = 2.00 (P = 0.05) |
|  | **Invasion** | Pharmacological inhibition | BT459 (3) | -5.78 [-12.04, 0.47] | Mean Difference (IV, Random, 95% CI) | Tau² = 17.51; Chi² = 4.57, df = 2 (P = 0.10); I² = 56% | Z = 1.81 (P = 0.07) |
|  |  |  | MCF7 (1) | -9.50 [-18.82, -0.19] | Mean Difference (IV, Random, 95% CI) | NA | Z = 2.00 (P = 0.05) |

**Table S7.** Summary of meta-analysis showing non-significant association of tumour weight/ volume and overt metastasis in in vivo mice model with pharmacological and genetic modulation of TRAF 2/4/6

| **Outcome** | | **Intervention** | **Type of cell cultures (no. studies)** | **Subgroup (std.) mean difference (95% CI)** | **Overall (std.) mean difference (95% CI)** | **Statistical method** | **Test for heterogeneity** | **Test for overall effect** |
| --- | --- | --- | --- | --- | --- | --- | --- | --- |
| **TRAF6** | **Metastasis** | Genetic upregulation | Lung metastasis (2)  Liver metastasis (1) | 5.21 [-4.66, 15.08]  1.20 [0.23, 2.17] | 1.41 [-0.14, 2.96] | Std. Mean Difference (IV, Random, 95% CI) | Tau² = 1.06; Chi² = 6.64, df = 2 (P = 0.04); I² = 70% | Z = 1.79 (P = 0.07) |

**Table S8.** Summary of meta-analysis showing non-significant association between TRAF4 expression and poor survival in breast cancer patients.

| **Intervention** | **Outcome** | **Type of survival (no. studies)** | **Subgroup hazard ratio (95% CI)** | **Overall hazard ratio (95% CI)** | **Statistical method** | **Test for heterogeneity** | **Test for overall effect** |
| --- | --- | --- | --- | --- | --- | --- | --- |
| **TRAF4 expression** | HR (95%CI) | Kaplan-Meier survival analysis (3) | NA | 0.69 [0.02, 24.58] | Hazard Ratio (IV, Random, 95% CI) | Tau² = 6.23; Chi² = 15.33, df = 1 (P < 0.0001); I² = 93% | Z = 0.21 (P = 0.84) |

| **Study ID** | **Diagnosis** | | **Numbers of patients** | | **Consecutive patients** | | **TRAF judgment** | | **Data source** | | **Total (out of 10)** |
| --- | --- | --- | --- | --- | --- | --- | --- | --- | --- | --- | --- |
|  | Clear **(**☆☆**)** | Unclear **(**☆**)** | >100 (☆☆**)** | <100 **(**☆**)** | Yes (☆☆**)** | Unclear **(**☆**)** | Detailed criteria (☆☆**)** | No description **(**☆**)** | HR and 95%CI (☆☆**)** | Survival curve **(**☆**)** |  |
| Lin et al,. 2014 | ☆☆ | | ☆☆ | | ☆ | | ☆☆ | | ☆☆ | | ☆☆☆☆☆☆☆☆☆ (9) |
| Rezaeian et al,. 2017 | ☆☆ | | ☆☆ | | ☆ | | ☆☆ | | ☆☆ | | ☆☆☆☆☆☆☆☆☆ (9) |
| Zhang et al,. 2013 | ☆☆ | | ☆☆ | | ☆ | | ☆☆ | | ☆☆ | | ☆☆☆☆☆☆☆☆ (8) |
| Zhao et al,. 2015 | ☆☆ | | ☆ | | ☆ | | ☆☆ | | ☆ | | ☆☆☆☆☆☆☆ (7) |

**Table S9.** Quality assessment for human studies

≤5 stars indicate low quality; 6–7 stars indicate medium quality; 8–10 stars indicate high quality.

**Table S10.** PRISMA checklist for article.

| **Section and Topic** | **Item #** | **Checklist item** | **Location where item is reported (page #)** |
| --- | --- | --- | --- |
| **TITLE** | | | 1 |
| Title | 1 | Identify the report as a systematic review. |  |
| **ABSTRACT** | | |  |
| Abstract | 2 | See the PRISMA 2020 for Abstracts checklist. | 2 |
| **INTRODUCTION** | | |  |
| Rationale | 3 | Describe the rationale for the review in the context of existing knowledge. | 3 |
| Objectives | 4 | Provide an explicit statement of the objective(s) or question(s) the review addresses. | 4 |
| **METHODS** | | |  |
| Eligibility criteria | 5 | Specify the inclusion and exclusion criteria for the review and how studies were grouped for the syntheses. | 4-5 |
| Information sources | 6 | Specify all databases, registers, websites, organisations, reference lists and other sources searched or consulted to identify studies. Specify the date when each source was last searched or consulted. | 4 |
| Search strategy | 7 | Present the full search strategies for all databases, registers and websites, including any filters and limits used. | Supplementary Table S1 |
| Selection process | 8 | Specify the methods used to decide whether a study met the inclusion criteria of the review, including how many reviewers screened each record and each report retrieved, whether they worked independently, and if applicable, details of automation tools used in the process. | 5-6 |
| Data collection process | 9 | Specify the methods used to collect data from reports, including how many reviewers collected data from each report, whether they worked independently, any processes for obtaining or confirming data from study investigators, and if applicable, details of automation tools used in the process. | 5-6 |
| Data items | 10a | List and define all outcomes for which data were sought. Specify whether all results that were compatible with each outcome domain in each study were sought (e.g. for all measures, time points, analyses), and if not, the methods used to decide which results to collect. | 5, Supplementary Tables S3-S5 |
|  | 10b | List and define all other variables for which data were sought (e.g. participant and intervention characteristics, funding sources). Describe any assumptions made about any missing or unclear information. | Supplementary Tables S3-S5 |
| Study risk of bias assessment | 11 | Specify the methods used to assess risk of bias in the included studies, including details of the tool(s) used, how many reviewers assessed each study and whether they worked independently, and if applicable, details of automation tools used in the process. | 6-7 |
| Effect measures | 12 | Specify for each outcome the effect measure(s) (e.g. risk ratio, mean difference) used in the synthesis or presentation of results. | Tables 2-4, Supplementary Tables 6-8 |
| Synthesis methods | 13a | Describe the processes used to decide which studies were eligible for each synthesis (e.g. tabulating the study intervention characteristics and comparing against the planned groups for each synthesis (item #5)). | 5 |
|  | 13b | Describe any methods required to prepare the data for presentation or synthesis, such as handling of missing summary statistics, or data conversions. | 6 |
|  | 13c | Describe any methods used to tabulate or visually display results of individual studies and syntheses. | 6 |
|  | 13d | Describe any methods used to synthesize results and provide a rationale for the choice(s). If meta-analysis was performed, describe the model(s), method(s) to identify the presence and extent of statistical heterogeneity, and software package(s) used. | 6 |
|  | 13e | Describe any methods used to explore possible causes of heterogeneity among study results (e.g. subgroup analysis, meta-regression). | 6 |
|  | 13f | Describe any sensitivity analyses conducted to assess robustness of the synthesized results. | NA |
| Reporting bias assessment | 14 | Describe any methods used to assess risk of bias due to missing results in a synthesis (arising from reporting biases). | 6-7 |
| Certainty assessment | 15 | Describe any methods used to assess certainty (or confidence) in the body of evidence for an outcome. | 7 |
| **RESULTS** | | |  |
| Study selection | 16a | Describe the results of the search and selection process, from the number of records identified in the search to the number of studies included in the review, ideally using a flow diagram. | 8, Figure 1 |
|  | 16b | Cite studies that might appear to meet the inclusion criteria, but which were excluded, and explain why they were excluded. | Supplementary Table S2 |
| Study characteristics | 17 | Cite each included study and present its characteristics. | Supplementary Tables S3-S5 |
| Risk of bias in studies | 18 | Present assessments of risk of bias for each included study. | 10, Supplementary Tables S9, Supplementary Figures S1-S2 |
| Results of individual studies | 19 | For all outcomes, present, for each study: (a) summary statistics for each group (where appropriate) and (b) an effect estimate and its precision (e.g. confidence/credible interval), ideally using structured tables or plots. | Tables 2-4, Supplementary tables S6-S8 |
| Results of syntheses | 20a | For each synthesis, briefly summarise the characteristics and risk of bias among contributing studies. | 11-15 |
|  | 20b | Present results of all statistical syntheses conducted. If meta-analysis was done, present for each the summary estimate and its precision (e.g. confidence/credible interval) and measures of statistical heterogeneity. If comparing groups, describe the direction of the effect. | 11-16 |
|  | 20c | Present results of all investigations of possible causes of heterogeneity among study results. | NA |
|  | 20d | Present results of all sensitivity analyses conducted to assess the robustness of the synthesized results. | NA |
| Reporting biases | 21 | Present assessments of risk of bias due to missing results (arising from reporting biases) for each synthesis assessed. | NA |
| Certainty of evidence | 22 | Present assessments of certainty (or confidence) in the body of evidence for each outcome assessed. | 11 |
| **DISCUSSION** | | |  |
| Discussion | 23a | Provide a general interpretation of the results in the context of other evidence. | 18-19 |
|  | 23b | Discuss any limitations of the evidence included in the review. | 21-22 |
|  | 23c | Discuss any limitations of the review processes used. | 21-22 |
|  | 23d | Discuss implications of the results for practice, policy, and future research. | 19-22 |
| **OTHER INFORMATION** | | |  |
| Registration and protocol | 24a | Provide registration information for the review, including register name and registration number, or state that the review was not registered. | 23 |
|  | 24b | Indicate where the review protocol can be accessed, or state that a protocol was not prepared. | 23 |
|  | 24c | Describe and explain any amendments to information provided at registration or in the protocol. | NA |
| Support | 25 | Describe sources of financial or non-financial support for the review, and the role of the funders or sponsors in the review. | 22 |
| Competing interests | 26 | Declare any competing interests of review authors. | 23 |
| Availability of data, code and other materials | 27 | Report which of the following are publicly available and where they can be found: template data collection forms; data extracted from included studies; data used for all analyses; analytic code; any other materials used in the review. | 22-23 |

**Table S11.** PRISMA checklist for abstract.

| **Section and Topic** | **Item #** | **Checklist item** | **Reported (Yes/No)** |
| --- | --- | --- | --- |
| **TITLE** | | |  |
| Title | 1 | Identify the report as a systematic review. | Yes |
| **BACKGROUND** | | |  |
| Objectives | 2 | Provide an explicit statement of the main objective(s) or question(s) the review addresses. | NO |
| **METHODS** | | |  |
| Eligibility criteria | 3 | Specify the inclusion and exclusion criteria for the review. | NO |
| Information sources | 4 | Specify the information sources (e.g. databases, registers) used to identify studies and the date when each was last searched. | YES |
| Risk of bias | 5 | Specify the methods used to assess risk of bias in the included studies. | NO |
| Synthesis of results | 6 | Specify the methods used to present and synthesise results. | YES |
| **RESULTS** | | |  |
| Included studies | 7 | Give the total number of included studies and participants and summarise relevant characteristics of studies. | YES |
| Synthesis of results | 8 | Present results for main outcomes, preferably indicating the number of included studies and participants for each. If meta-analysis was done, report the summary estimate and confidence/credible interval. If comparing groups, indicate the direction of the effect (i.e. which group is favoured). | YES |
| **DISCUSSION** | | |  |
| Limitations of evidence | 9 | Provide a brief summary of the limitations of the evidence included in the review (e.g. study risk of bias, inconsistency and imprecision). | YES |
| Interpretation | 10 | Provide a general interpretation of the results and important implications. | YES |
| **OTHER** | | |  |
| Funding | 11 | Specify the primary source of funding for the review. | NO |
| Registration | 12 | Provide the register name and registration number. | NA |

**Table S12.** KEGG enrichment analysis of TRAF2, 4, 6.

| **#term ID** | **term description** | **observed gene count** | **false discovery rate** | **matching proteins in your network (labels)** |
| --- | --- | --- | --- | --- |
| hsa04657 | IL-17 signaling pathway | 3 | 3.72E-05 | TRAF2,TRAF4,TRAF6 |
| hsa05222 | Small cell lung cancer | 3 | 3.72E-05 | TRAF2,TRAF4,TRAF6 |
| hsa05200 | Pathways in cancer | 3 | 0.0021 | TRAF2,TRAF4,TRAF6 |
| hsa04622 | RIG-I-like receptor signaling pathway | 2 | 0.0034 | TRAF2,TRAF6 |
| hsa04064 | NF-kappa B signaling pathway | 2 | 0.0055 | TRAF2,TRAF6 |
| **hsa04380** | **Osteoclast differentiation** | **2** | **0.0067** | **TRAF2,TRAF6** |
| hsa05135 | Yersinia infection | 2 | 0.0067 | TRAF2,TRAF6 |
| hsa05160 | Hepatitis C | 2 | 0.0081 | TRAF2,TRAF6 |
| hsa04621 | NOD-like receptor signaling pathway | 2 | 0.009 | TRAF2,TRAF6 |
| hsa05130 | Pathogenic Escherichia coli infection | 2 | 0.0093 | TRAF2,TRAF6 |
| hsa05131 | Shigellosis | 2 | 0.0093 | TRAF2,TRAF6 |
| hsa05132 | Salmonella infection | 2 | 0.0093 | TRAF2,TRAF6 |
| hsa05169 | Epstein-Barr virus infection | 2 | 0.0093 | TRAF2,TRAF6 |
| hsa05170 | Human immunodeficiency virus 1 infection | 2 | 0.0093 | TRAF2,TRAF6 |
| hsa04010 | MAPK signaling pathway | 2 | 0.0146 | TRAF2,TRAF6 |
| hsa05168 | Herpes simplex virus 1 infection | 2 | 0.0374 | TRAF2,TRAF6 |

**Table S13.** GO enrichment of TRAF2, 4, 6.

| **#term ID** | **term description** | **observed gene count** | **false discovery rate** | **matching proteins in your network (labels)** |
| --- | --- | --- | --- | --- |
| GO:0035631 | CD40 receptor complex | 2 | 0.0021 | TRAF2,TRAF6 |
| GO:0031996 | Thioesterase binding | 3 | 9.44E-07 | TRAF2,TRAF4,TRAF6 |
| GO:0005164 | Tumor necrosis factor receptor binding | 3 | 7.76E-06 | TRAF2,TRAF4,TRAF6 |
| GO:0031435 | Mitogen-activated protein kinase kinase kinase binding | 2 | 0.002 | TRAF2,TRAF6 |
| GO:0031625 | Ubiquitin protein ligase binding | 3 | 0.002 | TRAF2,TRAF4,TRAF6 |
| GO:0019901 | Protein kinase binding | 3 | 0.0152 | TRAF2,TRAF4,TRAF6 |
| GO:0008270 | Zinc ion binding | 3 | 0.0232 | TRAF2,TRAF4,TRAF6 |
| GO:0007250 | Activation of nf-kappab-inducing kinase activity | 3 | 1.17E-05 | TRAF2,TRAF4,TRAF6 |
| GO:0070534 | Protein k63-linked ubiquitination | 3 | 7.81E-05 | TRAF2,TRAF4,TRAF6 |
| GO:0033209 | Tumor necrosis factor-mediated signaling pathway | 3 | 0.00059 | TRAF2,TRAF4,TRAF6 |
| GO:0046330 | Positive regulation of jnk cascade | 3 | 0.00066 | TRAF2,TRAF4,TRAF6 |
| GO:0043122 | Regulation of i-kappab kinase/nf-kappab signaling | 3 | 0.002 | TRAF2,TRAF4,TRAF6 |
| GO:0051023 | Regulation of immunoglobulin secretion | 2 | 0.0024 | TRAF2,TRAF6 |
| GO:0002726 | Positive regulation of t cell cytokine production | 2 | 0.0027 | TRAF2,TRAF6 |
| GO:0032743 | Positive regulation of interleukin-2 production | 2 | 0.0056 | TRAF2,TRAF6 |
| GO:0007249 | I-kappaB kinase/NF-kappaB signaling | 2 | 0.0183 | TRAF2,TRAF6 |
| GO:0043507 | Positive regulation of jun kinase activity | 2 | 0.0183 | TRAF2,TRAF6 |
| GO:0051865 | Protein autoubiquitination | 2 | 0.0183 | TRAF2,TRAF6 |
| GO:0051092 | Positive regulation of nf-kappab transcription factor activity | 2 | 0.0439 | TRAF2,TRAF6 |

1. **Supplementary figures**

**Figure S 1.** Risk of bias (RoB) assessment for in vivo studies using the SYRCLE RoB tool. (a) Representative summary Table for the risk of bias assessment. Green cells with ‘+’ designate low risk of bias, yellow cells with ‘?’ designate unclear risk of bias, and red cells with ‘-’ designate high risk of bias. (b) Representative summary of risk of bias analysis across studies.

**Figure S2**. Risk of bias (RoB) assessment for in vitro studies using the OHAT RoB tool.

(a) Representative summary Table for the risk of bias assessment. Dark green cells with ‘++’ designate definitely low risk of bias, light green cells with ‘+’ designate low risk of bias, light pink cells with ‘-’designate probably high risk of bias, and yellow cells with ‘?’ designate unclear risk of bias. (b) Representative summary of risk of bias analysis across studies. In bold and italics are the articles that consist of in vivo and in vitro experiments and their quality was assessed with both tools (for in vivo and in vitro studies).

1. **Supplementary reference**

1 Brincas, H. M. *et al.* A genetic variant in microrna-146a is associated with sporadic breast cancer in a southern brazilian population. *Genetics and Molecular Biology* **42** (2019). https://doi.org:10.1590/1678-4685-GMB-2019-0278

2 Bilir, C., Engin, H., Can, M., Temi, Y. B. & Demirtas, D. The prognostic role of inflammation and hormones in patients with metastatic cancer with cachexia. *Medical Oncology* **32** (2015). https://doi.org:10.1007/s12032-015-0497-y

3 Chan, C.-H. *et al.* The Skp2-SCF E3 ligase regulates Akt ubiquitination, glycolysis, herceptin sensitivity, and tumorigenesis. *Cell* **149**, 1098-1111 (2012). https://doi.org:https://dx.doi.org/10.1016/j.cell.2012.02.065

4 He, J. *et al.* Integrative analysis of genomic amplification-dependent expression and loss-of-function screen identifies ASAP1 as a driver gene in triple-negative breast cancer progression. *Oncogene* **39**, 4118-4131 (2020). https://doi.org:10.1038/s41388-020-1279-3

5 Helbig, G. *et al.* NF-kappa B promotes breast cancer cell migration and metastasis by inducing the expression of the chemokine receptor CXCR4. *Journal of Biological Chemistry* **278**, 21631-21638 (2003). https://doi.org:10.1074/jbc.M300609200

6 Li, L., Chen, X. P. & Li, Y. J. MicroRNA-146a and human disease. *Scandinavian Journal of Immunology* **71**, 227-231 (2010). https://doi.org:10.1111/j.1365-3083.2010.02383.x

7 Li, Z. *et al.* Methylation of EZH2 by PRMT1 regulates its stability and promotes breast cancer metastasis. *Cell death and differentiation* **27**, 3226-3242 (2020). https://doi.org:https://dx.doi.org/10.1038/s41418-020-00615-9

8 Moelans, C. B. *et al.* Genomic evolution from primary breast carcinoma to distant metastasis: Few copy number changes of breast cancer related genes. *Cancer letters* **344**, 138-146 (2014). https://doi.org:https://dx.doi.org/10.1016/j.canlet.2013.10.025

9 Niu, M. M. *et al.* Noncanonical TGF-beta signaling leads to FBXO3-mediated degradation of Delta Np63 alpha promoting breast cancer metastasis and poor clinical prognosis. *Plos Biology* **19** (2021). https://doi.org:10.1371/journal.pbio.3001113

10 Patel, N. M. *et al.* Paclitaxel sensitivity of breast cancer cells with constitutively active NF-kappa B is enhanced by I kappa B alpha super-repressor and parthenolide. *Oncogene* **19**, 4159-4169 (2000). https://doi.org:10.1038/sj.onc.1203768

11 Qi, J. L. *et al.* SQSTM1/p62 regulate breast cancer progression and metastasis by inducing cell cycle arrest and regulating immune cell infiltration. *Genes and Diseases* (2021). https://doi.org:10.1016/j.gendis.2021.03.008

12 Sato, Y. *et al.* Possible Roles of Proinflammatory Signaling in Keratinocytes Through Aryl Hydrocarbon Receptor Ligands for the Development of Squamous Cell Carcinoma. *Frontiers in Immunology* **11** (2020). https://doi.org:10.3389/fimmu.2020.534323

13 Song, L. *et al.* Sinomenine inhibits breast cancer cell invasion and migration by suppressing NF-kappaB activation mediated by IL-4/miR-324-5p/CUEDC2 axis. *Biochemical and biophysical research communications* **464**, 705-710 (2015). https://doi.org:https://dx.doi.org/10.1016/j.bbrc.2015.07.004

14 Sun, Y. *et al.* Jatrorrhizine inhibits mammary carcinoma cells by targeting TNIK mediated Wnt/beta-catenin signalling and epithelial-mesenchymal transition (EMT). *Phytomedicine : international journal of phytotherapy and phytopharmacology* **63**, 153015 (2019). https://doi.org:https://dx.doi.org/10.1016/j.phymed.2019.153015

15 Tomasetto, C. *et al.* TRAF-4 expression in breast carcinomas [1] (multiple letters). *American Journal of Pathology* **153**, 2007-2008 (1998). https://doi.org:10.1016/S0002-9440(10)65714-8

16 van Dam, P. A., Verhoeven, Y. & Trinh, X. B. in *Tumor Microenvironment: Molecular Players, Pt B* Vol. 1277 *Advances in Experimental Medicine and Biology* (ed A. Birbrair) 53-62 (2020).

17 Bishop, R. T. *et al.* Combined administration of a small-molecule inhibitor of TRAF6 and Docetaxel reduces breast cancer skeletal metastasis and osteolysis. *Cancer Letters* **488**, 27-39 (2020). https://doi.org:10.1016/j.canlet.2020.05.021

18 Choi, J. M., Devkota, S., Sung, Y. H. & Lee, H. W. EI24 regulates epithelial-to-mesenchymal transition and tumor progression by suppressing TRAF2-mediated NF-κB activity. *Oncotarget* **4**, 2383-2396 (2013). https://doi.org:10.18632/oncotarget.1434

19 Jang, K. W. *et al.* Ubiquitin Ligase CHIP Induces TRAF2 Proteasomal Degradation and NF-kappa B Inactivation to Regulate Breast Cancer Cell Invasion. *Journal of Cellular Biochemistry* **112**, 3612-3620 (2011). https://doi.org:10.1002/jcb.23292

20 Jiang, L. *et al.* MiR-892b silencing activates NF-kB and promotes aggressiveness in breast cancer. *Cancer Research* **76**, 1101-1111 (2016). https://doi.org:10.1158/0008-5472.CAN-15-1770

21 Li, Z. *et al.* Plumbagin inhibits breast tumor bone metastasis and osteolysis by modulating the tumor-bone microenvironment. *Current Molecular Medicine* **12**, 967-981 (2012). https://doi.org:10.2174/156652412802480871

22 Liu, R. H. *et al.* FOXP3 Controls an miR-146/NF-kappa B Negative Feedback Loop That Inhibits Apoptosis in Breast Cancer Cells. *Cancer Research* **75**, 1703-1713 (2015). https://doi.org:10.1158/0008-5472.Can-14-2108

23 Liu, J. H. *et al.* The MyD88 inhibitor TJ-M2010-2 suppresses proliferation, migration and invasion of breast cancer cells by regulating MyD88/GSK-3 beta and MyD88/NF-kappa B signalling pathways. *Experimental Cell Research* **394** (2020). https://doi.org:10.1016/j.yexcr.2020.112157

24 Peramuhendige, P. *et al.* TRAF2 in osteotropic breast cancer cells enhances skeletal tumour growth and promotes osteolysis. *Scientific Reports* **8** (2018). https://doi.org:10.1038/s41598-017-18327-5

25 Shi, D. *et al.* TLR5: A prognostic and monitoring indicator for triple-negative breast cancer. *Cell Death and Disease* **10** (2019). https://doi.org:10.1038/s41419-019-2187-8

26 Wang, X., Jin, C., Tang, Y., Tang, L. Y. & Zhang, Y. E. Ubiquitination of tumor necrosis factor receptor-associated factor 4 (TRAF4) by smad ubiquitination regulatory factor 1 (Smurf1) regulates motility of breast epithelial and cancer cells. *Journal of Biological Chemistry* **288**, 21784-21792 (2013). https://doi.org:10.1074/jbc.M113.472704

27 Wang, S., Feng, X., Wang, Y., Li, Q. & Li, X. Dysregulation of tumour microenvironment driven by circ-TPGS2/miR-7/TRAF6/NF-κB axis facilitates breast cancer cell motility. *Autoimmunity* **54**, 284-293 (2021). https://doi.org:10.1080/08916934.2021.1931843

28 Yao, Y. *et al.* Wogonoside inhibits invasion and migration through suppressing TRAF2/4 expression in breast cancer. *Journal of experimental & clinical cancer research : CR* **36**, 103 (2017). https://doi.org:https://dx.doi.org/10.1186/s13046-017-0574-5

29 Zhang, L. *et al.* TRAF4 promotes TGF-beta receptor signaling and drives breast cancer metastasis. *Molecular cell* **51**, 559-572 (2013). https://doi.org:https://dx.doi.org/10.1016/j.molcel.2013.07.014

30 Zheng, T. *et al.* CXCR4 3'UTR functions as a ceRNA in promoting metastasis, proliferation and survival of MCF-7 cells by regulating miR-146a activity. *European Journal of Cell Biology* **94**, 458-469 (2015). https://doi.org:10.1016/j.ejcb.2015.05.010

31 Lin, Y. *et al.* Functional role of asparaginyl endopeptidase ubiquitination by TRAF6 in tumor invasion and metastasis. *Journal of the National Cancer Institute* **106** (2014). https://doi.org:10.1093/jnci/dju012

32 Rezaeian, A. H. *et al.* A hypoxia-responsive TRAF6-ATM-H2AX signalling axis promotes HIF1 alpha activation, tumorigenesis and metastasis. *Nature Cell Biology* **19**, 38-51 (2017). https://doi.org:10.1038/ncb3445

33 Zhu, L. Y., Zhang, S. S., Huan, X. J., Mei, Y. & Yang, H. W. Down-regulation of TRAF4 targeting RSK4 inhibits proliferation, invasion and metastasis in breast cancer xenografts. *Biochemical and Biophysical Research Communications* **500**, 810-816 (2018). https://doi.org:10.1016/j.bbrc.2018.04.164

34 Zhao, Z.-J. *et al.* Expression, correlation, and prognostic value of TRAF2 and TRAF4 expression in malignant plural effusion cells in human breast cancer. *Diagnostic cytopathology* **43**, 897-903 (2015). https://doi.org:https://dx.doi.org/10.1002/dc.23330
